# Carbon source–dependent transcriptomic regulation and monosaccharide remodeling of exopolysaccharide biosynthesis in *Pediococcus pceusentosa* LL-07

**DOI:** 10.1186/s12866-025-04409-2

**Published:** 2025-10-28

**Authors:** Kuan Lu, Xueya Wang, Ying Zhou, Qiujin Zhu

**Affiliations:** 1 Guizhou Province Key Laboratory of Agricultural and Animal Products Storage and Processing, School of Liquor and Food Engineering, Guizhou University, Guiyang, Guizhou 550025 China; 2 Guizhou Biotechnology Research and Development Base Co., Ltd, Guiyang, Guizhou 550025 China; 3 Chili Pepper Research Institute, Guizhou Provincial Academy of Agricultural Sciences, Guiyang, Guizhou 550006 China

**Keywords:** *Pediococcus pentosaceus*, Transcriptomics, Exopolysaccharide, Comparative genomics

## Abstract

**Background:**

Exopolysaccharides (EPSs) produced by lactic acid bacteria (LAB) play important roles in industrial applications. The type of carbon source affects both the production and composition of EPSs. However, the molecular mechanisms underlying this regulation in *Pediococcus pentosaceus* remain poorly understood. This study explores the effects of different carbon sources on the EPS biosynthesis pathway and monosaccharide composition in *P. pentosaceus* LL-07, with particular emphasis on transcriptional regulation.

**Results:**

EPS yields were similar under glucose, fructose, or lactose (*p >* 0.05). However, RNA-Seq revealed distinct gene expression patterns. KEGG and GO analyses showed activation of carbohydrate metabolism and EPS-related pathways. Lactose significantly upregulated genes in the Leloir pathway (*galK*, *galT*, *galE*), nucleotide sugar biosynthesis (*pgm*, *galU*), and key EPS cluster genes (*wzz*, *wzc*, *gt0590*). This suggests extensive transcriptional remodeling. Monosaccharide analysis of purified EPS showed that all EPSs mainly contained glucose, galactose, and mannose. However, their composition varied with the carbon source: EPSs from lactose and glucose had more galactose, while fructose-derived EPSs contained more mannose. This matched the transcriptional data. GEPS-1 exhibits better in vitro antioxidant activity and thermogravimetric properties.

**Conclusion:**

Carbon source selection alters the transcriptional profile of *P. pentosaceus* LL-07, affecting the monosaccharide composition, in vitro antioxidant activity, and thermogravimetric properties of its EPSs. These findings connect sugar metabolism to EPS structural changes. They provide a potential strategy to tailor microbial polysaccharides through substrate engineering for applications in functional foods and biotechnology.

**Supplementary Information:**

The online version contains supplementary material available at 10.1186/s12866-025-04409-2.

## Background

Exopolysaccharides (EPSs) produced by lactic acid bacteria (LAB) are high molecular weight carbohydrate polymers. They play key roles in bacterial responses to environmental stress, biofilm formation, and host colonization [1]. From a biotechnological viewpoint, EPS attract considerable research interest because they improve the texture, viscosity, and stability of fermented foods [2, 3]. Moreover, EPS exhibit bioactive properties such as antioxidant, immunomodulatory, and prebiotic effects [4–6]. The physical, chemical, and biological properties of EPS closely relate to their chemical structures [7, 8]. These structures are influenced by the producing strains, environmental conditions, and substrate availability [9, 10].

A key factor affecting EPS yield and composition is the type of carbon source [10]. Cerning et al. [11] found that EPS produced by *Lactobacillus casei* CG11 varied in yield and monosaccharide composition under different carbon sources. Angelov et al. [12] studied EPS and biomass accumulation in test strains using glucose, fructose, and sucrose. They found sucrose was best for EPS production in most strains, but glucose led to the highest biomass. Strains made similar EPS levels with glucose and fructose, but fructose was less good for biomass. Valerio et al. [13] investigated EPS synthesis in 21 lactic acid bacteria strains, which were cultured on agar media containing sucrose, fructose, or glucose as carbohydrate sources. Results showed that only 8 *Weissella cibaria*, 2 *Weissella confusa*, and 2 *Leuconostoc* strains could synthesize EPS using sucrose. The remaining strains were unable to utilize these carbon sources for EPS synthesis. Different sugars are assimilated via distinct transport systems and metabolic pathways. These systems influence gene expression related to central carbon metabolism and EPS biosynthesis [14, 15]. In LAB, EPS biosynthesis relies on enzymes encoded in specific gene clusters. These include glycosyltransferases, glycoribonucleotide biosynthesis enzymes, and chain-length regulators [16, 17]. These clusters regulate processes such as the addition of monosaccharide residues to repeating units, polymerisation, and secretion of the final EPS product. EPS structural diversity among LAB species results from variations in the metabolism of biosynthetic clusters and precursors [18]. *Pediococcus pentosaceus*, a member of LAB, has been shown to function as a fermenter and bio-preservative, thereby enhancing flavour and safety of food products [19–21]. Some *P. pentosaceus* strains produce neutral or heteropolysaccharide EPSs, which have excellent functional properties [22, 23]. *P. pentosaceus* LL-07, isolated from Suanrou (Libo, Guizhou, China), is a potential probiotic. Whole-genome analysis revealed a complete EPS gene cluster, indicating its ability to synthesise EPS [24]. However, the genetic and metabolic mechanisms behind EPS production in *P. pentosaceus* LL-07, particularly its response to different carbon sources, remain unclear.

This study integrates transcriptome analysis, EPS quantification, and structural characterization to identify carbon source-specific regulatory pathways and their effects on EPS yield and monosaccharide composition. The findings enhance understanding of how carbon flux is linked to EPS biosynthesis in LAB. They provide new insights into tailoring EPS properties via fermentation substrate engineering.

## Materials and methods

### Strain and culture conditions

*P. pentosaceus* LL-07 was activated by three successive transfers in de Man Rogosa Sharpe (MRS) broth (Shanghai Bio-way Technology Co., Ltd., China). The activated culture was adjusted to an OD value of 1. It was then inoculated into sugar-free MRS (SF-MRS) liquid medium. This medium was supplemented with 20 g/L of a single carbon source—either glucose, fructose, lactose, maltose, or sucrose. The culture was incubated at 37 °C for 48 h. Experimental groups were designated as G, F, L, M, and S, corresponding to the respective carbon sources. Samples collected during the early logarithmic growth phases (OD_600_ between 0.2 and 0.3) and initial stationary growth phases (OD_600_ between 0.8 and 0.9) were labeled as −1 and − 2, respectively. EPS content was measured at the initial stationary phase using the phenol-sulfuric acid method [25]. All inoculations were carried out in MRS-based media under identical conditions to ensure comparability among treatments.

### RNA extraction and sequencing

Total RNA was extracted from *P. pentosaceus* LL-07 using an RNA extraction kit (Sangon Biotech, Shanghai, China). RNA quality was assessed using a NanoDrop One UV–Vis microvolume spectrophotometer (Thermo Scientific, USA). RNA libraries were constructed using the Illumina^®^ Stranded mRNA Prep Ligation Kit (Illumina, USA). Paired-end RNA sequencing (RNA-seq) was performed on the Illumina NovaSeq 6000 platform (Illumina, USA). Sequencing reads were aligned to the Rfam database to calculate the proportion of ribosomal RNA (rRNA) using its annotations. An rRNA content below 15% is generally considered acceptable for subsequent analyses. RNA integrity number (RIN, 1–10) was determined using a RiboCop Kit (Lexogen, USA) on an Agilent 5300 Fragment Analyzer (Agilent, USA), with higher values indicating better integrity.

### Bioinformatics and differential expression analysis

Filtered reads were aligned to the *P. pentosaceus* LL-07 reference genome using Bowtie2 (http://bowtie-bio.sourceforge.net/bowtie2/index.shtml). Gene function annotation was performed using multiple databases, including NCBI (https://www.ncbi.nlm.nih.gov/), UniProt (https://www.uniprot.org/), Gene Ontology (GO; http://geneontology.org/), and Kyoto Encyclopedia of Genes and Genomes (KEGG; https://www.kegg.jp/).

Gene expression levels were quantified with RSEM (http://deweylab.github.io/RSEM/) and expressed as fragments per kilobase of transcript per million mapped reads (FPKM). Pearson correlation coefficients were used to assess reproducibility among biological replicates. Principal component analysis (PCA) was performed using SIMCA^®^ software (version 14.1, Sartorius Stedim Data Analytics AB, Umeå, Sweden) to analyze differences in expression levels between samples.

Differential analysis of differentially expressed genes (DEGs) across treatment groups was conducted using DESeq2 (https://bioconductor.org/packages/release/bioc/html/DESeq2.html). To control the false discovery rate (FDR), p-values from statistical tests were adjusted using the Benjamini-Hochberg method. DEGs were filtered based on two criteria: |log_2_ fold change| >1 for expression changes and adjusted p-value < 0.05, as detected by DESeq2. Group comparisons are summarized in Table 1.

**Table 1:** Comparison of difference groups

Treatment vs. Control	Treatment vs. Control
G-2 vs. G-1	F-1 vs. L-1
F-2 vs. F-1	F-2 vs. G-2
L-2 vs. L-1	L-2 vs. G-2
F-1 vs. G-1	L-2 vs. F-2
L-1 vs. G-1	

Functional enrichment of differentially expressed genes (DEGs) in GO categories was performed using GOATOOLS [26], with significance assessed by Fisher’s exact test. Multiple testing corrections were applied using Holm, Bonferroni, false discovery rate (FDR), and Sidak methods. GO terms with an adjusted *p-*value < 0.05 were considered significantly enriched.

KEGG pathway enrichment analysis of DEGs was conducted using KOBAS 2.0 [27]. The same statistical correction methods were applied, and pathways with an adjusted *p*-value < 0.05 were considered significantly enriched.

### Quantitative real-time PCR (qRT-PCR)

qRT-PCR was used to validate a subset of randomly selected DEGs, thereby confirming the accuracy and reliability of the transcriptomic data. All reactions were performed in triplicate for each experimental group. Primer sequences used for qRT-PCR are listed in Supplementary Table 1.

### EPS extraction and purification

EPS was extracted following the method described by Lu et al. [24]. The fermentation broth was centrifuged at 10,000 ×*g* for 15 min at 4 °C to remove cells. The resulting supernatant was treated with trichloroacetic acid (TCA) to a final concentration of 4% (v/v) and subjected to protein removal using Sevag reagent. Following a second centrifugation, the supernatant was mixed with four volumes of chilled anhydrous ethanol (1:4, v/v) and incubated at 4 °C overnight to precipitate EPS. The precipitate was dialyzed in deionized water using a membrane with 8,000–14,000 Dalton cutoff, then lyophilized to obtain crude EPS. The crude EPS was dissolved in 0.05 mol/L Tris-HCl buffer to 10 mg/ml, purified via Cellulose DE-52 and Sepharose CL-6B columns, and finally purified EPS was obtained.

### Monosaccharide composition analysis

Take purified EPS samples (3 mg) and add 1 ml of 2 M TFA acid solution. Heat at 121 ℃ for 2 h. Blow dry with nitrogen gas. Add 99.99% methanol for washing, then blow dry again. The procedure was repeated three times to ensure complete removal of residual acid. Add sterile water to dissolve the sample, then transfer it to a chromatographic vial for subsequent analysis. Monosaccharide composition was analyzed using an ICS 5000 + ion chromatography system (Thermo Scientific, Waltham, MA, USA).

### In vitro antioxidant activity

The purified EPS was dissolved in distilled water to prepare sample solutions at concentrations of 0.25, 0.5, 1.0, 2.0, 4.0, and 8.0 mg/mL. For each purified EPS sample, the superoxide anion radical scavenging capacity [28], 2,2-diphenyl-1-picrylhydrazyl radical (DPPH) radical scavenging capacity [29], and hydroxyl radical scavenging capacity [30] were determined. Ascorbic acid (Vc) was used as a control, and the above procedures were repeated.

### Thermogravimetric analysis

2.0 mg of the purified EPS powder was placed in a crucible and heated from 25 °C to 800 °C at a heating rate of 20 °C/min to determine weight changes via thermogravimetric analysis (TG) and derivative thermogravimetric analysis (DTG) [31].

### Statistical analysis

All experiments were conducted in triplicate. Data are presented as mean ± standard deviation (SD). One-way ANOVA analysis was performed using SPSS 19.0 software (IBM Corp., Armonk, NY, USA), followed by Tukey’s HSD test.

## Results

### Growth characteristics and EPS production under different carbon sources

**Fig. 1:**
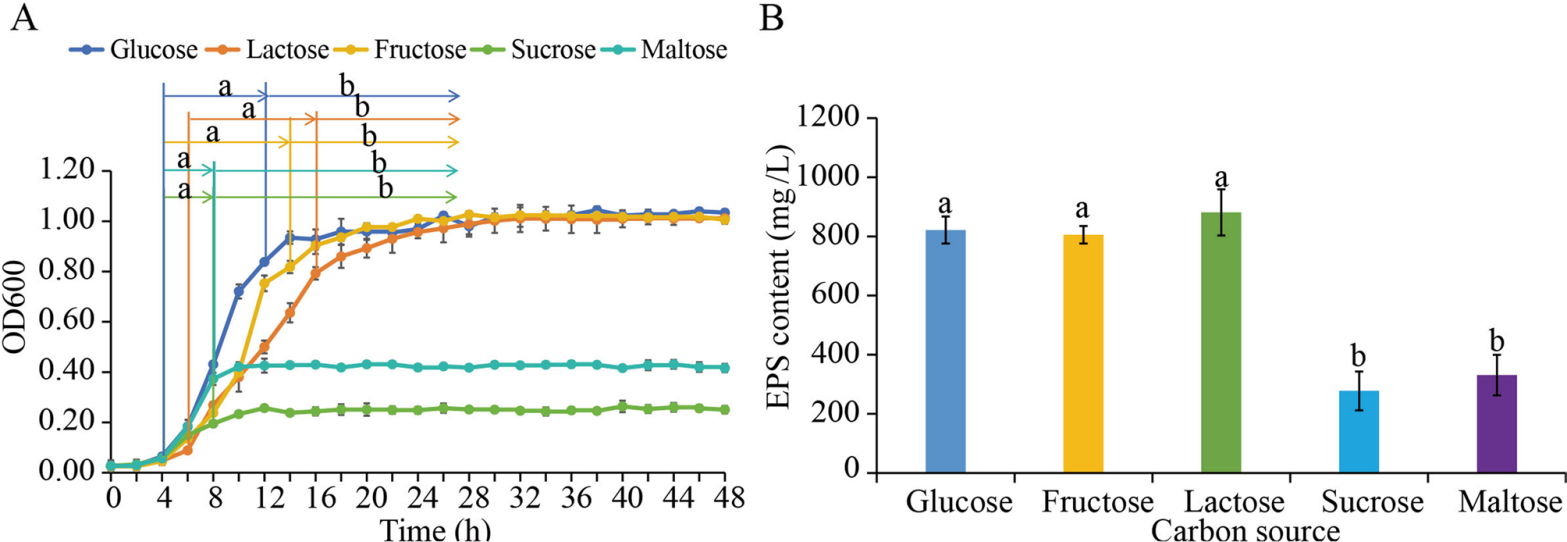
Growth curves and EPS content of *P. pentosaceus* LL-07 under different carbon source culture conditions. **A** Growth curves. a represents the logarithmic growth stage, and b indicates the entry into stable growth. **B** EPS content. The same lowercase letters indicate *p* > 0.05, while different lowercase letters indicate *p* < 0.05

*P. pentosaceus* LL-07 grew differently under different carbon source conditions (Fig. 1A). When sucrose or maltose was used as the carbon source, the strain entered the stationary phase after about 8 h. However, the overall cell density was relatively low, with the OD values of the bacterial cultures all below 0.5. In comparison, cultures with glucose, fructose, or lactose grew better. The time they took to reach the stationary phase was slightly different: about 12 h for glucose, 14 h for fructose, and 16 h for lactose. All three carbon sources led to high cell concentrations during the stationary phase, with the OD values of the bacterial cultures all around 1. These results show that *P. pentosaceus* LL-07 metabolizes glucose, fructose, and lactose more efficiently than sucrose and maltose.

EPS production by *P. pentosaceus* LL-07 varied depending on the carbon source (Fig. 1B). When sucrose and maltose were used, EPS yields were relatively low, at 277.58 ± 65.73 mg/L and 330.84 ± 68.69 mg/L, respectively. In contrast, cultures with glucose, fructose, or lactose showed significantly higher EPS production, with average yields of 821.47 ± 45.76 mg/L, 805.33 ± 29.58 mg/L, and 881.18 ± 77.92 mg/L, respectively. Although metabolic differences among these three sugars were apparent, EPS yields did not differ significantly (*p >* 0.05). These findings demonstrate that carbon source selection affects EPS biosynthesis in *P. pentosaceus* LL-07 and provide a basis for transcriptomic investigation into sugar-specific regulatory mechanisms.

### RNA-seq data quality and expression analysis

Global transcriptomic profiles of *P. pentosaceus* LL-07 cultured with three different carbon sources were analyzed using RNA-Seq. All raw sequencing libraries exhibited base error rates below 0.1% (Supplementary Table 2). After quality control and filtering, high-quality reads were obtained from 18 libraries (three biological replicates per sugar treatment), with Q20 and Q30 values exceeding 95% and 93%, respectively (Supplementary Table 3). These results confirmed that the sequencing data met the quality requirements for downstream analyses. Alignment to the *P. pentosaceus* LL-07 reference genome showed mapping rates above 97% for all groups (Supplementary Table 4). In addition, more than 94% of the clean reads in each group uniquely aligned to the reference genome, ensuring the reliability of subsequent transcriptomic analyses. Furthermore, the rRNA removal efficiency and RIN meet the requirements (Supplementary Table 5, Supplementary Fig. 1).

**Fig. 2:**
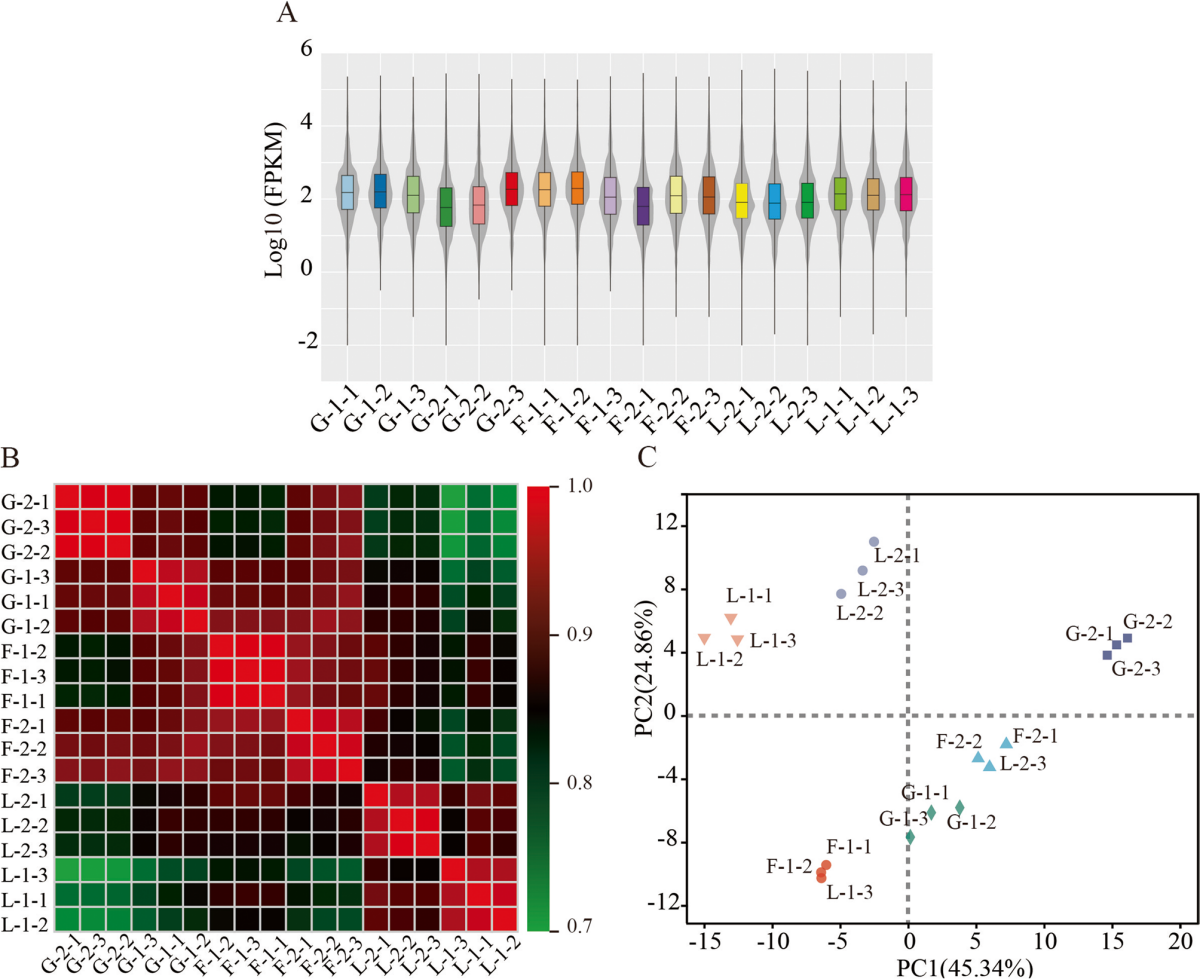
Graph of expression analysis between groups. **A** statistical plot of FPKM density distribution. Each color represents a sample. Expanded sections in the plot show where gene expression is most concentrated in the sample. **B** heat map of correlation analysis between groups. The redder the color, the higher the correlation. **C** PCA for each group

As shown in Fig. 2A, most genes have log₁₀(FPKM + 1) values between 0.5 and 3.5. Only a small number of genes show extremely high expression (log₁₀(FPKM + 1) > 4.5). Under each condition, biological replicates exhibit highly consistent distributions. This indicates good reproducibility of the RNA-Seq data and supports subsequent differential expression analyses. Pearson correlation coefficients between biological replicates exceeded 0.85 (Fig. 2B), indicating high experimental reproducibility. Principal component analysis (PCA) further revealed distinct separation of samples by carbon source, with L clustering separately from G and F (Fig. 2C). These findings suggest that, although growth profiles and EPS yields were similar among G, F, and L, the transcriptional response to lactose was notably different. Additionally, the tight clustering of biological replicates within each group further supports the reliability of the RNA-seq dataset.

### Differential gene expression analysis

**Fig. 3:**
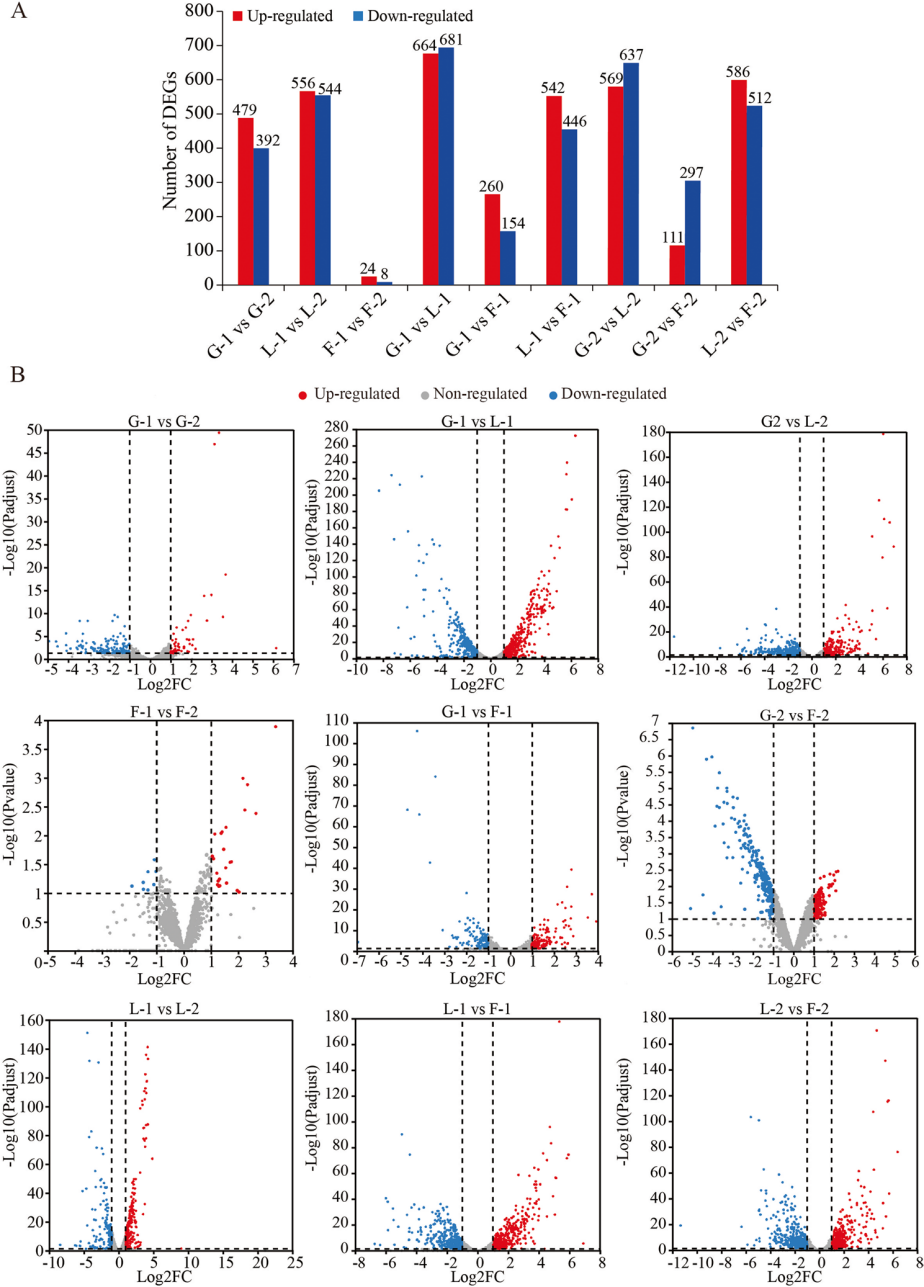
Statistical and volcano graphs of DEGs. **A** Statistical graphs. Bar plots showing the number of upregulated and downregulated DEGs across different comparisons. **B** Volcano graphs. Red dots represent upregulated genes, blue dots represent downregulated genes, and grey dots indicate genes with no significant expression change. Genes closer to zero on the x-axis exhibit lower fold changes in expression

Statistical analyses and volcano plots of differentially expressed genes (DEGs) (Fig. 3) revealed significant transcriptomic alterations in *P. pentosaceus* LL-07 between different growth phases under the same carbon source. Specifically, 479 genes were upregulated and 392 downregulated in the G-1 vs. G-2 comparison; 556 genes were upregulated and 544 downregulated in the L-1 vs. L-2 comparison; whereas only 24 genes were upregulated and 8 downregulated in the F-1 vs. F-2 comparison. These results suggest more extensive transcriptional reprogramming during the growth phase transition in glucose and lactose groups compared to fructose. Significant differences in gene expression were also observed between carbon sources at the same growth stage. In the G-1 vs. F-1 and G-1 vs. L-1 comparisons, 260 and 664 genes were upregulated, and 154 and 681 were downregulated, respectively. In L-1 vs. F-1, 542 genes were upregulated and 446 downregulated. At the stationary phase, comparisons of G-2 vs. F-2 and G-2 vs. L-2 revealed 111 and 569 upregulated genes, and 297 and 637 downregulated genes, respectively. In the L-2 vs. F-2 comparison, 586 genes were upregulated and 512 were downregulated.

These findings demonstrate that both the carbon source and the growth phase significantly affect the global gene expression profile of *P. pentosaceus* LL-07. Notably, the lactose group showed the most pronounced transcriptomic alterations, indicating that lactose metabolism may trigger broader metabolic and regulatory responses. This underscores the organism’s capacity to dynamically modulate its transcriptional networks in response to environmental changes.

### Functional enrichment of differentially expressed genes

**Fig. 4:**
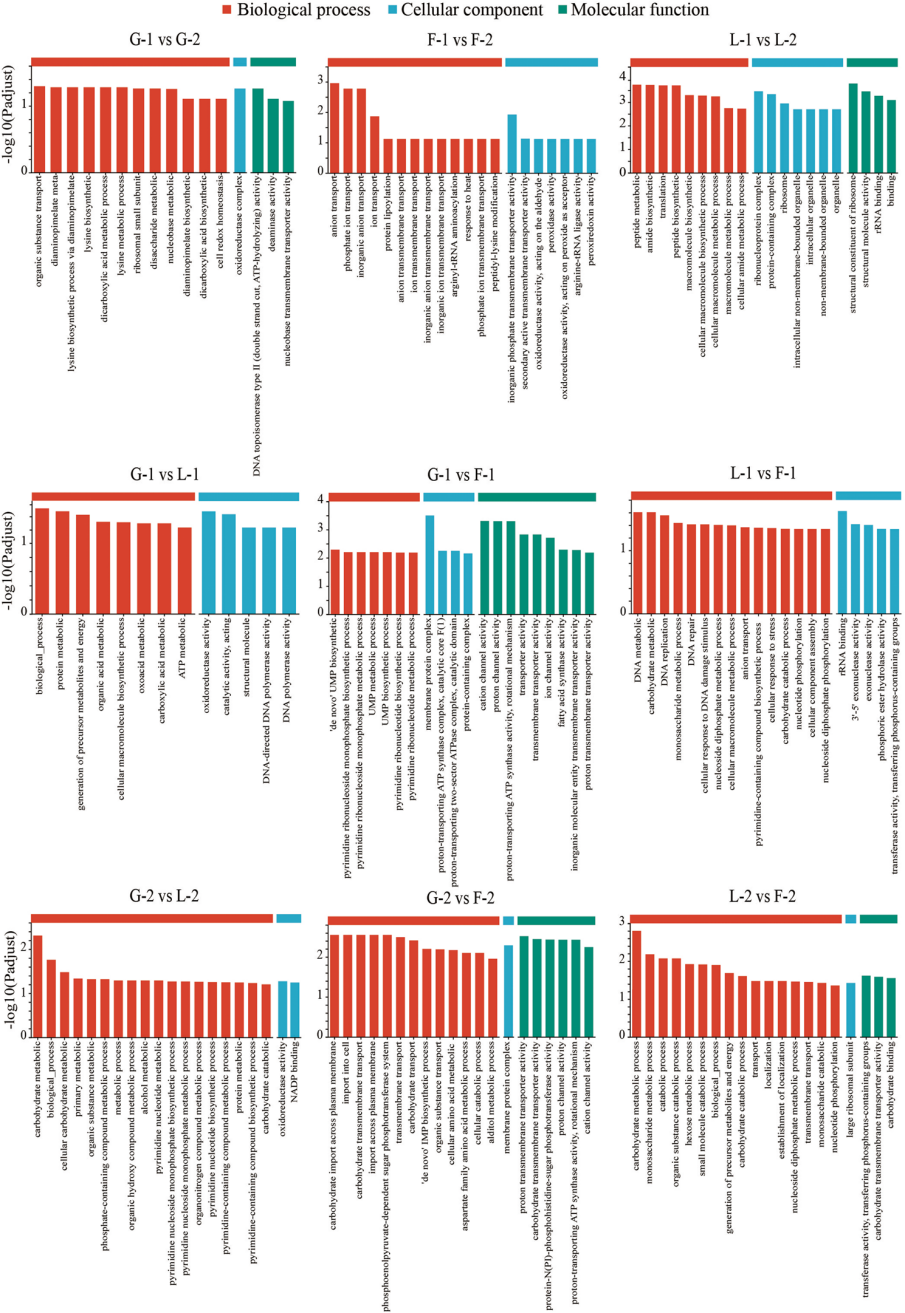
GO enrichment analysis. Only the first 20 pathways are displayed. Red indicates biological process, blue indicates cellular component, and green indicates molecular function

GO enrichment analysis revealed that most DEGs were significantly associated with biological processes (BP), followed by molecular functions (MF) and cellular components (CC) (Fig. 4). Time-course comparisons showed that lactose (L-2 vs. L-1) induced more extensive transcriptional changes than glucose or fructose, with enriched GO terms related to carbohydrate metabolism, sugar transport, and transcriptional regulation. Similarly, at the same time point, lactose-related comparisons (e.g., L-1 vs. F-1 or L-2 vs. F-2) showed a greater number of enriched GO terms than those based on glucose or fructose, indicating that lactose elicits more complex gene regulatory responses.

**Fig. 5:**
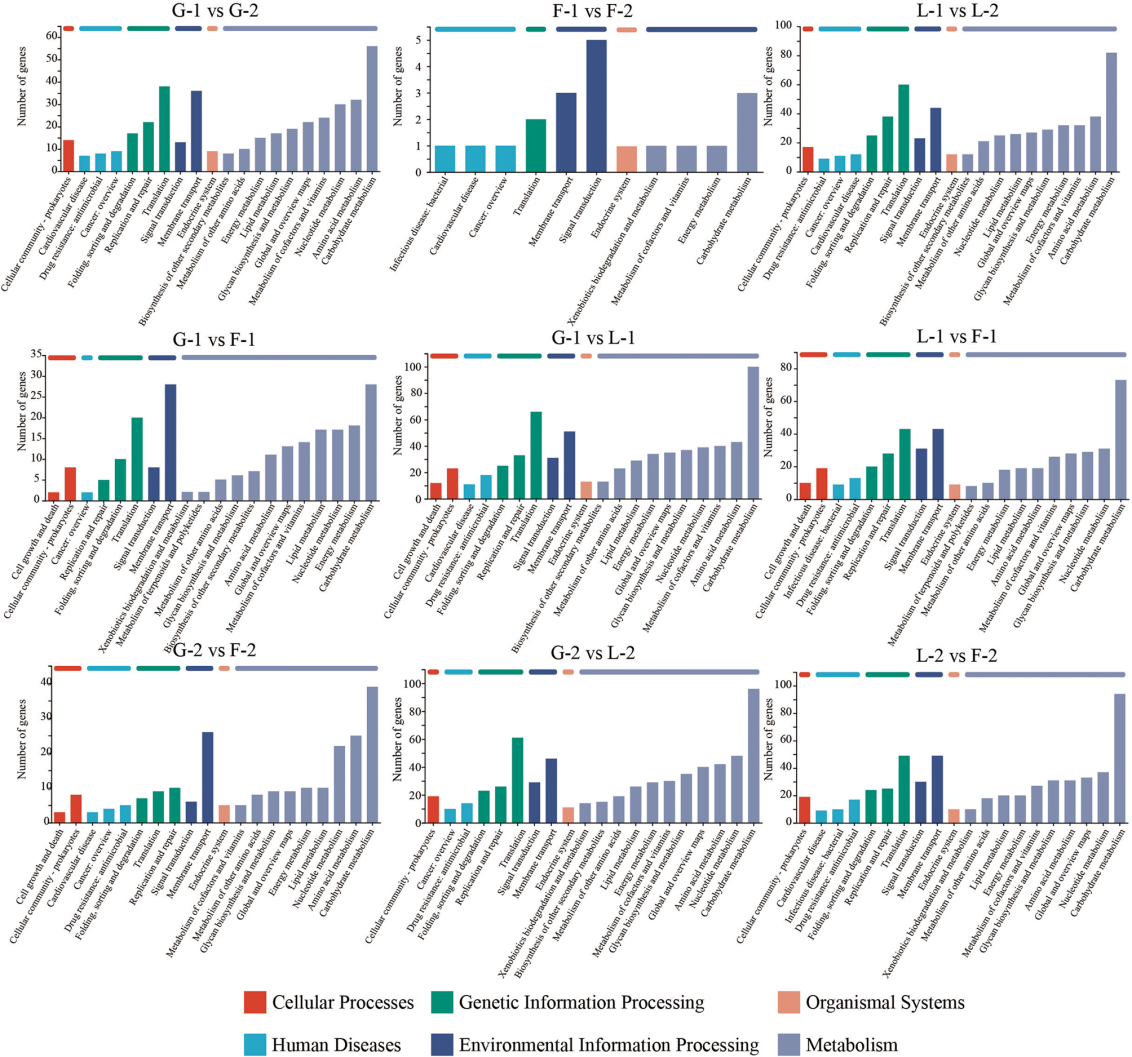
KEGG enrichment analysis. Only the first 20 pathways are displayed. The vertical axis represents the number of genes, and the horizontal axis represents the function

**Fig. 6:**
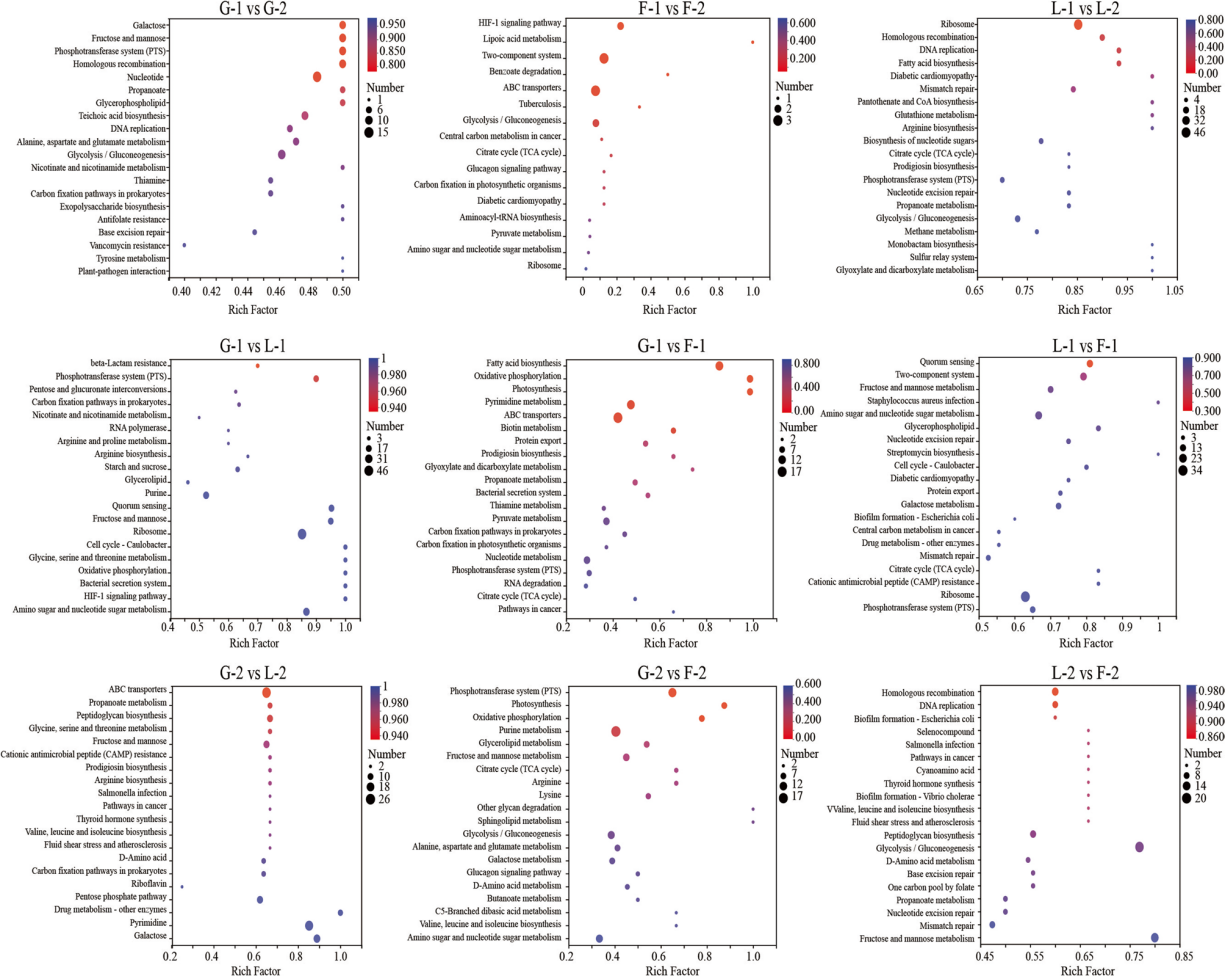
Bubble plot of KEGG. Only the first 20 pathways are displayed. The vertical axis represents the pathway name, and the horizontal axis represents the enrichment factor. The size of the bubbles represents the number of genes, and the color indicates Padjust

KEGG pathway enrichment further supported these findings. DEGs across all comparisons were primarily enriched in metabolic pathways, including carbohydrate metabolism, nucleotide metabolism, and glycan biosynthesis (Figs. 5 and 6). Notably, DEGs identified in lactose-related comparisons were enriched in amino sugar and nucleotide sugar metabolism, fructose and mannose metabolism, peptidoglycan biosynthesis, and glycolysis/gluconeogenesis—pathways closely associated with EPS biosynthesis and cell wall remodeling.

These enrichment results collectively indicate that both carbon source and growth phase significantly influence functional gene expression patterns in *P. pentosaceus* LL-07. Lactose, in particular, induces broader transcriptional and metabolic adaptations, consistent with its role in promoting distinct EPS biosynthetic pathways.

### Effects of different carbon sources on the EPS Synthesis Pathway

To further investigate the effects of different carbon sources on EPS synthesis in *P. pentosaceus* LL-07, samples from groups G-2, L-2, and F-2 were selected for pairwise comparisons. Genes related to the EPS metabolic pathway were identified among the DEGs of each group (Table 2), and their regulatory functions were annotated using GO and KEGG databases.

**Table 2:** Statistics of DEGs associated with EPS synthesis in the different groups

Groups	Gene ID	Gene Name	Putative Function	Log2FC	Regulate
L-2 vs. G-2	*gene0195*	*galK*	galactokinase	5.01	up
	*gene0196*	*galE*	UDP-glucose 4-epimerase	5.36	up
	*gene1424*	*galE*	UDP-glucose 4-epimerase	1.32	up
	*gene0197*	*galT*	UDP-glucose-hexose-1-phosphate uridylyltransferase	5.77	up
	*gene1391*	*scrK*	fructokinase	1.35	up
	*gene0429*	*galU*	UTP-glucose-1-phosphate uridylyltransferase	1.00	up
	*gene0431*	*pgm*	phosphoglucomutase	1.02	up
	*gene0578*	*wzz*	chain-length determining protein	1.77	up
	*gene0579*	*wzc*	CpsD/CapB family tyrosine-protein kinase	1.83	up
	*gene0580*	*-*	CpsB/CapC family capsule biosynthesis tyrosine phosphatase	1.79	up
	*gene0585*	*-*	Stealth CR1 domain-containing protein	1.35	up
	*gene0587*	*-*	polysaccharide polymerase	1.76	up
	*gene0588*	*glf*	UDP-galactopyranose mutase	1.75	up
	*gene0589*	*wzx*	polysaccharide biosynthesis C-terminal domain-containing protein	1.23	up
	*gene0590*	*gt0590*	glycosyltransferase	2.3	up
	*gene0320*	*nagB*	glucosamine-6-phosphate deaminase	-1.13	down
	*gene0996*	*murQ*	N-acetylmuramic acid 6-phosphate etherase	-2.93	down
	*gene1477*	*nagA*	N-acetylglucosamine 6-phosphate deacetylase	-1.17	down
	*gene0116*	*galK*	galactokinase	-1.01	down
F-2 vs. G-2	*gene0141*	*fruK*	1-phosphofructokinase	1.05	up
	*gene0580*	*cpsB*	CpsB/CapC family capsule biosynthesis tyrosine phosphatase	1.00	up
	*gene0996*	*murQ*	N-acetylmuramic acid 6-phosphate etherase	-1.77	down
	*gene0116*	*galK*	galactokinase	-1.08	down
	*gene0278*	*glmU*	bifunctional UDP-N-acetylglucosamine pyrophosphorylase / Glucosamine-1-phosphate N-acetyltransferase	-1.03	down
	*gene0608*	*wecB*	UDP-N-acetylglucosamine 2-epimerase (non-hydrolysing)	-1.16	down
L-2 vs. F-2	*gene0195*	*galK*	galactokinase	5.24	up
	*gene0196*	*galE*	UDP-glucose 4-epimerase	5.51	up
	*gene0197*	*galT*	UDPglucose–hexose-1-phosphate uridylyltransferase	5.64	up
	*gene0578*	*wzz*	chain-length determining protein	1.08	up
	*gene0590*	*gt0590*	glycosyltransferase	1.26	up
	*gene0320*	*nagB*	glucosamine-6-phosphate deaminase	-1.38	down
	*gene0996*	*murQ*	N-acetylmuramic acid 6-phosphate etherase	-1.24	down
	*gene1519*	*murA*	UDP-N-acetylglucosamine 1-carboxyvinyltransferase	-1.25	down

### Expression of sugar uptake and nucleotide-sugar biosynthesis genes

Transcriptomic analysis revealed that lactose significantly activated the Leloir pathway, which converts galactose into nucleotides sugar essential for EPS biosynthesis. Notably, *galK* was significantly upregulated (log₂FC: +5.01 in L-2 vs. G-2; +5.24 in L-2 vs. F-2), along with *galT* (+ 5.77 and + 5.64) and *galE* (+ 5.36 and + 5.51). These substantial increases suggest enhanced metabolic flux from galactose to UDP-Gal and UDP-Glc. This finding aligns with previous studies showing that *galT* overexpression promotes EPS production in *Lactobacillus* strains [32]. In addition to the Leloir pathway, *pgm* and *galU* were upregulated (log₂FC: +1.02 and + 1.00, respectively, in L-2 vs. G-2) under lactose conditions. Collectively, these genes contribute to the enhanced synthesis of UDP-glucose and UDP-galactose, key precursors for EPS polymerization.

In contrast, cells cultured with fructose showed suppressed expression of *galK*, *galT*, and *galE*, reflecting fructose phosphorylation and glycolytic entry, thereby bypassing the Leloir pathway. Additionally, genes involved in amino sugar metabolism (e.g., *nagA*, *nagB*, *murQ*, *glmU*) were slightly downregulated in the lactose group, suggesting reduced carbon flux toward peptidoglycan synthesis, potentially to prioritize EPS production.

### Activation of EPS cluster and glycosyltransferase genes

The 13-gene EPS biosynthetic cluster in *P. pentosaceus* LL-07 encodes genes involved in regulation, polymerization, and export [24]. Under lactose conditions, several cluster genes were significantly upregulated, including *wzz* (log₂FC + 1.77), *wzc* (+ 1.83), *cpsB* (+ 1.79), and *gt0590* (+ 2.30). Upregulation of *wzz*, which modulates polysaccharide chain length, may influence the physical and immunological properties of EPS [33]. The *gt0590*, putatively encoding a glycosyltransferase, is likely involved in galactose incorporation into EPS, based on its co-expression with *galK*, *galT*, and *galE*. Additionally, *wzx* (log₂FC + 1.23) and *glf* (+ 1.75) were upregulated, supporting the assembly and export of complex heteropolysaccharides.

### qRT-PCR

**Fig. 7:**
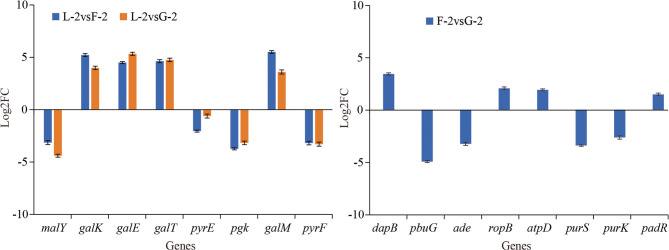
The results of qRT-PCR. The area above the horizontal axis indicates gene upregulation, while the area below represents gene downregulation

To validate the RNA-Seq results, eight DEGs were randomly selected from the L-2 vs. G-2, F-2 vs. G-2, and L-2 vs. F-2 comparisons for qRT-PCR analysis (Fig. 7, Supplementary Fig. 6, Supplementary Fig. 7). The results showed expression patterns consistent with the RNA-Seq data, confirming the reliability of the transcriptomic analysis.

### EPS monosaccharide composition analysis

EPSs produced from glucose (GEPS), fructose (FEPS), and lactose (LEPS) cultures were purified and analyzed using ultraviolet–visible (UV–Vis) spectroscopy and ion chromatography. All samples exhibited strong absorbance in the 190–210 nm range, indicating the presence of polysaccharides, and showed no absorbance at 260–280 nm, confirming the absence of nucleic acid and protein contamination (Supplementary Fig. 2).

The composition of extracellular polysaccharides synthesized by *P. pentosaceus* LL-07 varied under different carbon source cultures. After purification, GEPS and LEPS each contained three types of polysaccharides, whereas FEPS consisted of two. Monosaccharide composition analysis revealed that GEPS, FEPS, and LEPS were primarily composed of mannose, glucose, and galactose, which together accounted for more than 97% of the total sugar content (Supplementary Fig. 5, Table 3). However, their relative abundances differed: GEPS and LEPS were rich in galactose, while FEPS contained a higher proportion of mannose. The absence of uronic acids suggests that these EPSs are neutral heteropolysaccharides [34].

**Table 3:** Monosaccharide composition of EPS

Sample ID	Monosaccharide (%)					
	Fuc	Ara	Gal	Glc	Man	Rib
GEPS	0.98	0.38	25.42	21.62	50.57	0.75
LEPS	0.52	0.30	27.45	18.65	52.28	0.59
FEPS	0.57	1.03	7.15	25.86	64.35	0.00

In vitro **antioxidant activity**

### In vitro antioxidant activity

As shown in Supplementary Fig. 3, all polysaccharides from different carbon source fermentations exhibited antioxidant activity, with variations among them. Among these, FEPS-1 had relatively weaker activity than other polysaccharides, while GEPS-1 showed prominent in vitro antioxidant activity. For GEPS-1, the half-inhibitory concentrations (IC₅₀) for hydroxyl radical, DPPH radical, and superoxide anion scavenging were 3.83, 4.45, and 3.35 mg/mL, respectively (Supplementary Table 6), indicating superior in vitro antioxidant activity.

### Thermogravimetric analysis

Thermogravimetric analysis of purified polysaccharides showed significant thermal property differences among those from different carbon source fermentations (Supplementary Fig. 4). In 25–100 °C, FEPS-1 had the most obvious weight loss, decreasing by 11.96%. In 100–300 °C, GEPS-2, LEPS-1, LEPS-3, and FEPS-1 lost weight sharply, with decreases of 44.87%, 48.90%, 38.36%, and 40.15%, respectively. GEPS-3, LEPS-2, and FEPS-2 stayed relatively stable within 0–300 °C. Notably, GEPS-1 remained stable throughout heating, with only 17.34% weight loss at the end of the experiment.

## Discussion

**Fig. 8:**
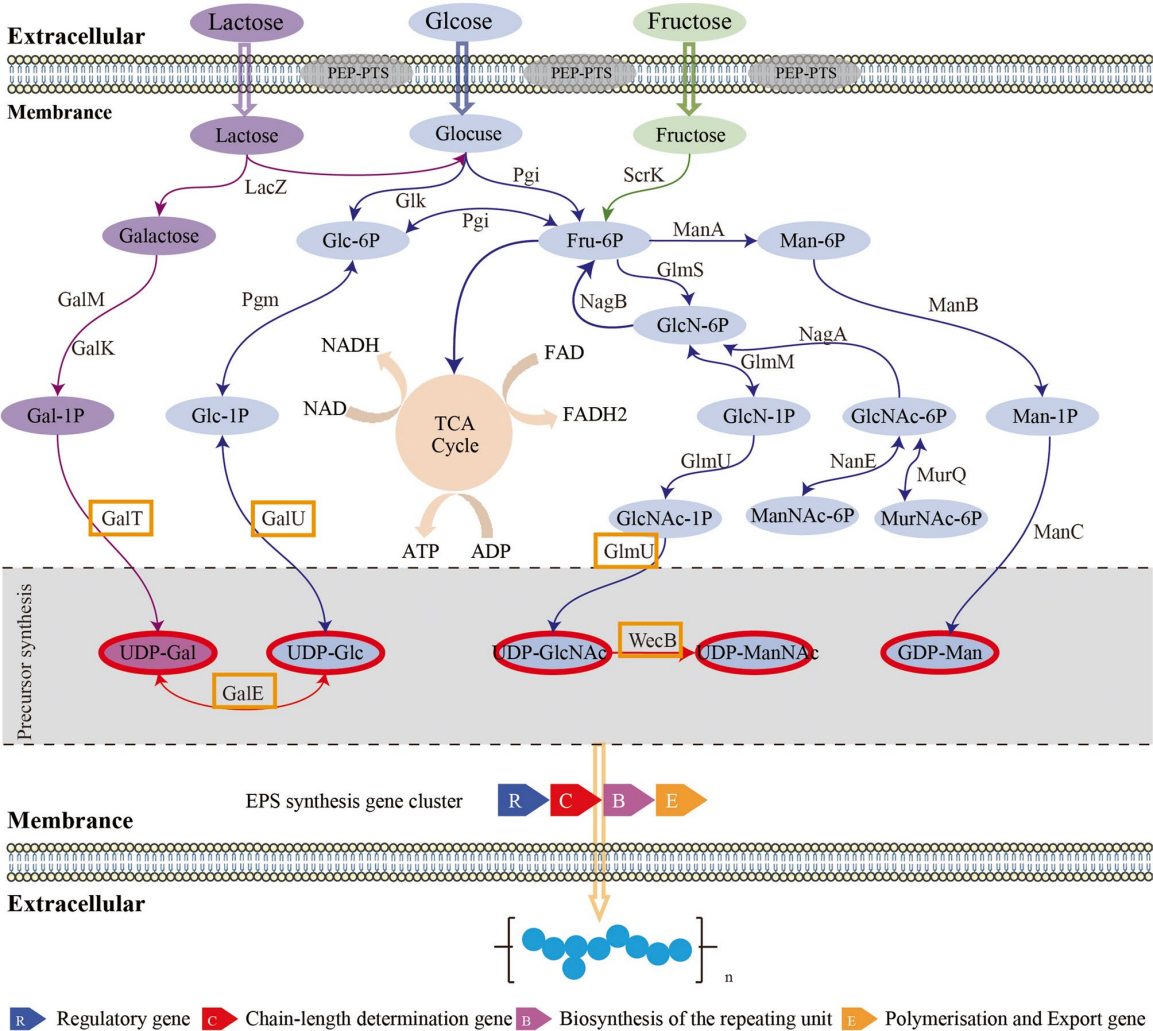
The EPS synthetic pathway of Pediococcus pentosaceus LL-07. Arrows indicate the direction of carbon flux. Enzymes highlighted in yellow boxes are key enzymes regulating carbon flux. Key intermediates are shown in red ovals. Enzymes and their EC numbers are as follows: LacZ, beta-galactosidase [EC:3.2.1.23]; GalM, aldose 1-epimerase [EC:5.1.3.3]; GalK, galactokinase [EC:2.7.1.6]; GalT, UDPglucose–hexose-1-phosphate uridylyltransferase [EC:2.7.7.12]; GalE, UDP-glucose 4-epimerase [EC:5.1.3.2]; GalU, UTP-glucose-1-phosphate uridylyltransferase [EC:2.7.7.9]; Pgm, phosphoglucomutase [EC:5.4.2.2]; Glk, glucokinase [EC:2.7.1.2]; Pgi, glucose-6-phosphate isomerase [EC:5.3.1.9]; ScrK, fructokinase [EC:2.7.1.4]; ManA, mannose-6-phosphate isomerase [EC:5.3.1.8]; GlmS, glutamine-fructose-6-phosphate transaminase (isomerizing) [EC:2.6.1.16]; NagB, glucosamine-6-phosphate deaminase [EC:3.5.99.6]; NagA, N-acetylglucosamine-6-phosphate deacetylase [EC:3.5.1.25]; GlmM, phosphoglucosamine mutase [EC:5.4.2.10]; GlmU, bifunctional UDP-N-acetylglucosamine pyrophosphorylase/Glucosamine-1-phosphate N-acetyltransferase [EC:2.7.7.23; 2.3.1.157]; NanE, N-acylglucosamine-6-phosphate 2-epimerase [EC:5.1.3.9]; MurQ, N-acetylmuramic acid 6-phosphate etherase [EC:4.2.1.126]; WecB, UDP-N-acetylglucosamine 2-epimerase (non-hydrolysing) [EC:5.1.3.14]

In this study, we investigated how different carbon sources—glucose, fructose, and lactose—influence the transcriptional regulation and monosaccharide composition of EPS biosynthesis in *P. pentosaceus* LL-07. By integrating RNA-Seq, functional enrichment analysis, and EPS monosaccharide composition, we demonstrated that carbon source selection modulates central metabolic pathways and directs carbon flux toward specific nucleotide-sugar precursors and EPS biosynthetic pathways (Fig. 8).

### Carbon-source–dependent transcriptional reprogramming

Transcriptomic profiling revealed that *P. pentosaceus* LL-07 exhibits distinct global gene expression patterns when cultured with glucose, fructose, or lactose, despite similar growth dynamics and EPS yields across these conditions (Fig. 1A, B). Principal component analysis (Fig. 2C) showed that cells grown on lactose formed a separate cluster from those on glucose or fructose, indicating broader transcriptional remodeling. Differential expression analysis further confirmed that lactose induced the greatest number of DEGs, whereas fructose induced the fewest (Fig. 3A). This suggests that lactose metabolism involves more extensive regulatory adjustments, likely due to the activation of the Leloir pathway and related transport systems [35]. Lactose is typically taken up via phosphotransferase system (PTS) components or lactose permeases, then hydrolyzed by *β*-galactosidase into glucose and galactose, which enter different metabolic routes [36, 37]. Consistent with this, we observed strong upregulation of Leloir pathway genes. Similar coordinated induction has been reported in *Lactobacillus plantarum* grown on lactose-containing media [38]. However, compared with lactic acid bacteria such as *Lactobacillus* and *Streptococcus*, *P. pentosaceus* exhibits unique characteristics in the regulation of EPS synthesis gene clusters. For example, the EPS synthesis gene cluster of *P. pentosaceus* includes 13 genes, such as glycosyltransferases, chain length regulators, tyrosine kinases, and phosphatases, whereas EPS gene clusters in *Lactobacillus* and *Streptococcus* differ in composition and structure [24].

By contrast, fructose enters glycolysis without involving the PTS or galactose metabolism, which likely accounts for the relatively modest transcriptional shifts observed [39]. Growth phase comparisons also revealed extensive transcriptomic remodeling during the transition from logarithmic (–1) to early stationary (–2) phase in both the glucose (479 upregulated, 392 downregulated DEGs) and lactose groups (556 upregulated, 544 downregulated DEGs). In contrast, fructose-grown cells showed minimal changes (24 upregulated, 8 downregulated DEGs). These findings suggest that fructose supports a metabolically stable state across growth phases, potentially due to its straightforward integration into central metabolism [40].

Notably, the widespread transcriptional changes triggered by lactose may reflect its dual function as both a nutrient and a regulatory signal [41]. Our findings support a model in which lactose initiates a coordinated transcriptional response that integrates carbon source sensing with activation of energy metabolism, precursor biosynthesis, and EPS assembly, thereby linking environmental cues to EPS production [42].

### Regulation of nucleotide sugar precursor synthesis

EPS biosynthesis critically depends on nucleotide-activated sugar precursors, including UDP-glucose (UDP-Glc), UDP-galactose (UDP-Gal), UDP-N-acetylglucosamine (UDP-GlcNAc), and GDP-mannose (GDP-Man). Transcriptomic data revealed that lactose markedly upregulated key genes involved in the synthesis of these precursors, including *galK*, *galT*, *galE*, *pgm*, and *galU* (Table 2; Fig. 8). GalK phosphorylates galactose to produce galactose-1-phosphate, initiating the Leloir pathway, whereas GalT catalyzes the transfer of the galactosyl group to UDP, forming UDP-Gal [43]. The upregulation of these genes likely increases metabolic flux toward both UDP-Gal and UDP-Glc, facilitating galactose incorporation and glucose recycling. Pgm catalyzes the reversible conversion of glucose-1-phosphate (Glc-1P) and glucose-6-phosphate (Glc-6P), directing carbon flux toward either glycolysis or EPS precursor synthesis [44]. Its upregulation in lactose-grown cells indicates a metabolic shift favoring Glc-1P utilization for UDP-Glc synthesis via GalU [45]. Previous studies in *Streptococcus thermophiles* have demonstrated that co-overexpression of *pgm* and *galU* significantly enhances heteropolysaccharide production, indicating their potential in metabolic engineering [46]. Interestingly, genes involved in amino sugar metabolism—including *nagA*, *nagB*, and *murQ*—were downregulated under lactose conditions. These genes typically function in recycling peptidoglycan-derived intermediates, such as GlcNAc-6P and MurNAc-6P, back into glycolysis [47]. Their downregulation may preserve these intermediates for cell wall synthesis and redirect metabolic flux toward EPS precursor production, illustrating how carbon sources influence metabolic prioritization.

Together, these results reveal a carbon source–dependent transcriptional strategy in *P. pentosaceus* LL-07. Lactose triggers robust activation of sugar nucleotide biosynthesis to ensure an adequate supply of EPS precursors, whereas glucose and fructose rely on more direct glycolytic routes with relatively modest transcriptional changes. These findings provide a foundation for targeted metabolic engineering to optimize EPS production and tailor its composition in lactic acid bacteria.

### EPS gene cluster activation and chain-length regulation

The EPS biosynthetic gene cluster in *P. pentosaceus* LL-07 comprises 13 genes that encode glycosyltransferases, chain-length regulators, tyrosine kinases and phosphatases, flippases, and export-related proteins [24]. Transcriptomic analysis revealed significant upregulation of several cluster genes in lactose-grown cells. These include *wzz* (log₂FC + 1.77), *wzc* (+ 1.83), *cpsB* (+ 1.79), and *gt0590* (+ 2.30) (Table 2; Fig. 8). Wzz family proteins regulate the modal length of polysaccharide chains, influencing the rheological and functional properties of EPS. In *Escherichia coli* and *Salmonella*, Wzz controls the O-antigen chain length, which affects virulence and immune evasion [48, 49]. In LAB, Wzz homologs are less studied. However, they are thought to modulate EPS chain length [50], influencing viscosity, mouthfeel, and texture in fermented dairy products [51]. The upregulation of *wzz* in lactose-grown cells suggests a transcriptional adjustment. This may help optimize EPS physicochemical traits under this carbon source. The kinase-phosphatase pair *wzc* and *cpsB* forms a regulatory module that controls EPS polymerization. Wzc is a membrane-bound tyrosine kinase that autophosphorylates to promote polymer elongation. CpsB (or its homolog CapC) dephosphorylates Wzc, which modulates chain termination and export [52]. This phosphorylation cycle is essential for tightly regulated control of polysaccharide length and structure. In *Streptococcus pneumoniae*, loss of tyrosine phosphatase function leads to abnormal capsule synthesis. This underscores the importance of this regulatory system [53]. The coordinated upregulation of *wzc* and *cpsB* in *P. pentosaceus* LL-07 suggests a fine-tuned EPS polymerization mechanism. This mechanism appears responsive to lactose availability. Gt0590 is likely involved in adding specific monosaccharide residues, possibly galactose, to the elongating EPS chain. Its expression correlates with upregulation of *galK*, *galT*, and *galE*. This supports its role in lactose-driven EPS biosynthesis. In *Lactobacillus casei* strains, glycosyltransferases have been implicated in EPS biosynthesis [54]. Similarly, expressing glycosyltransferases in other LAB has produced structurally novel glucans [31]. Wzx transports lipid-linked oligosaccharide repeat units across the cytoplasmic membrane. This is a key step in EPS polymerization and export [55]. Its moderate upregulation (+ 1.23) under lactose conditions suggests higher EPS production activity. The elevated expression of *glf* (+ 1.75) also indicates possible incorporation of UDP-Gal into the EPS.

Beyond transcriptional regulation, several post-transcriptional mechanisms influence EPS biosynthesis. In many Gram-positive and Gram-negative bacteria, EPS clusters encode a conserved tyrosine kinase–phosphatase module (Wzc/Wze and CpsB/Wzb), which regulates polymerization and export via reversible phosphorylation cycles. In *Lactobacillus rhamnosus*, tyrosine phosphorylation of Wze acts as a molecular switch controlling its interaction with the modulator protein Wzd, thereby modulating EPS chain assembly independently of transcript levels [56]. In *Streptococcus pneumoniae*, phosphorylation of the CpsD/Wze kinase and dephosphorylation by the CpsB phosphatase are essential for capsule production and proper spatial control during cell division [57, 58]. Similarly, in *Escherichia coli*, the phosphorylation state of Wzc determines its oligomerization and interaction with export machinery, directly affecting exopolysaccharide yield and chain length [52]. These findings suggest protein stability, kinase autophosphorylation, and phosphatase activity can fine-tune EPS production, even with modest gene expression changes. Although we did not experimentally evaluate post-transcriptional regulation in this study, coordinated upregulation of *wzc* and *cpsB* in *P. pentosaceus* LL-07 strongly implies phosphorylation-dependent control may operate alongside transcriptional responses to lactose. Future studies combining transcriptomic, proteomic, and phosphoproteomic approaches will help dissect how these post-transcriptional layers shape EPS output.

Another limitation of this study is the lack of quantitative analysis of the molecular weight (Mw) or Mw distribution of EPS. Mw is a key determinant of the rheological properties and texture of LAB EPS—high Mw and more linear polymers typically confer higher viscosity and gel strength in dairy matrices [59]. Additionally, carbon sources can alter the molecular weight distribution of EPS (e.g., high-Mw components were detected when strains were cultured using lactose or sucrose as carbon sources) [60]. Mechanistically, the modal chain length in the Wzy-dependent system is regulated by the Wzz family and influenced by the phosphorylation status/oligomerization of the tyrosine kinase Wzc [52]. Therefore, the upregulation of wzz and wzc observed under lactose conditions suggests a potential shift in chain length/Mw distribution, which requires direct measurement for verification. Future studies will determine the Mw of EPS using methods such as SEC-MALS or AF4-MALS and correlate it with wzz/wzc expression levels and the material properties of GEPS/LEPS/FEPS.

Overall, these results show that *P. pentosaceus* LL-07 uses a substrate-responsive regulatory program. This program integrates EPS precursor synthesis, chain-length regulation, and export. Lactose triggers coordinated activation of genes involved in both nucleotide sugar synthesis and polysaccharide assembly. Such regulation may allow the bacterium to fine-tune EPS structure and properties in response to the available carbon source. This adaptation may influence texture, stability, and bioactivity in functional food applications.

### Functional enrichment: linking metabolism to EPS assembly

Functional enrichment analysis (GO and KEGG) revealed that DEGs under different carbon sources were involved in pathways connecting central carbohydrate metabolism to EPS biosynthesis. Under lactose conditions, GO enrichment highlighted terms such as “carbohydrate metabolic process,” “nucleotide-sugar biosynthetic process,” and “polysaccharide biosynthetic process” (Fig. 4). These findings suggest a transcriptional emphasis on sugar conversion and EPS polymer assembly. Enrichment in “transmembrane transporter activity” and “phosphotransferase system” further highlights the key role of sugar uptake in shaping intracellular metabolism. KEGG pathway analysis (Fig. 5) supported these results. It showed significant enrichment in pathways such as “fructose and mannose metabolism”, “amino sugar and nucleotide sugar metabolism”, “peptidoglycan biosynthesis”, and “glycolysis/gluconeogenesis”. The “amino sugar and nucleotide sugar metabolism” pathway includes enzymes like GlmS, GlmM, GlmU, NagA, and NanE. These enzymes catalyze the conversion of Glc-6P derivatives into nucleotide sugars, including UDP-GlcNAc and UDP-ManNAc [61]. These activated sugars function in both peptidoglycan and EPS biosynthesis, highlighting a key metabolic branch point regulated by gene expression and carbon source availability. Under lactose, *nagA* and *nagB* were downregulated, while *glmU* was upregulated. This result suggests a metabolic shift favoring EPS precursor synthesis over amino sugar recycling. Although GlcNAc was not detected in the final EPS product (Table 2; Fig. 8), transcription still favored GlcNAc biosynthesis pathways. This result suggests a cellular intent to maintain precursor availability for potential polysaccharide integration or cell wall remodeling. Enrichment of the “peptidoglycan biosynthesis” pathway further indicates that carbon source availability influences both EPS metabolism and cell envelope biogenesis. Peptidoglycan turnover produces intermediates such as MurNAc-6P and GlcNAc-6P, which are typically recycled through the actions of MurQ and NagA/NagB. The observed repression of *murQ*, *nagA*, and *nagB* under lactose (Table 2; Fig. 8) implies a regulatory shift that conserves amino sugar intermediates for synthetic pathways rather than catabolic recycling. Additionally, the “fructose and mannose metabolism” pathway was enriched under both lactose and fructose conditions, aligning with the central role of fructose-6-phosphate and mannose-6-phosphate as metabolic branch-point intermediates. Mannose-6-phosphate is a direct precursor to GDP-mannose, another nucleotide sugar incorporated into EPS [62]. While our study primarily focused on UDP-glucose and UDP-galactose, the detection of mannose residues in EPS (Table 3) confirms the active utilization of GDP-mannose in polysaccharide assembly.

Collectively, these enrichment analyses underscore the extensive metabolic coordination linking carbon source utilization to EPS precursor biosynthesis, polymerization gene expression, and cell wall component synthesis. Our findings demonstrate how carbon sources modulate gene expression at both metabolic and structural levels. These insights provide a framework for metabolic engineering to improve EPS yield and structure in *P. pentosaceus*.

### Monosaccharide remodeling: structural consequences of transcriptional shifts

Monosaccharide analysis of purified EPS (GEPS, FEPS, and LEPS) showed that all three are neutral heteropolysaccharides mainly composed of mannose, glucose, and galactose. Together, these sugars account for over 97% of total content (Table 3). However, FEPS was clearly distinguished by its markedly higher mannose proportion, in contrast to GEPS and LEPS which were enriched in galactose. This indicates a carbon source-dependent remodeling of EPS structures: lactose promoted galactose incorporation, while fructose strongly favored mannose enrichment. These carbon-dependent shifts in monosaccharide ratios are not only compositional but also functional. Galactose-enriched EPSs, such as GEPS and LEPS, have been reported to exert stronger immunomodulatory effects, including the promotion of dendritic cell maturation and increased secretion of interleukin-10 (IL-10) [63]. These effects are often attributed to specific glycosidic linkages and branching patterns introduced by particular glycosyltransferases (GTs) [64]. The transcriptional upregulation of *gt0590* in response to lactose further suggests its role in directing galactose incorporation into EPS backbones, potentially reorganizing intermolecular hydrogen bonds and altering the supramolecular structure of the polymers. In contrast, FEPS was uniquely characterized by a marked enrichment in mannose. Mannose-rich EPSs are often linked to enhanced adhesion to mucosal surfaces via interactions with mannose-binding lectins [65]. This functional property likely arises from fructose-induced upregulation of genes involved in GDP-mannose biosynthesis, providing abundant substrate for mannose-specific GTs and favoring the assembly of mannose-rich polymers. Thus, the distinct mannose enrichment in FEPS highlights a carbon source-specific remodeling pathway that differs fundamentally from the galactose-dominated EPS observed under lactose or glucose conditions. Interestingly, although transcriptomic data indicated increased GlcNAc biosynthesis under lactose, no GlcNAc was detected in the final EPS. This gap between gene expression and EPS composition suggests that EPS GTs may have low affinity for UDP-GlcNAc, or that GlcNAc precursors are used mainly for peptidoglycan synthesis. This interpretation is supported by the GO and KEGG enrichment data indicating a repression of *nagA*, *nagB* and *murQ*, and upregulation of *glmU*, potentially skewing amino sugar utilization toward cell wall maintenance (Fig. 4–Fig. 6). Moreover, the absence of uronic acids (e.g., glucuronic acid, galacturonic acid) in all EPS variants indicates that the polymers are neutral, rather than acidic. Neutral EPS can form stable gel networks under acidic conditions, particularly in dairy matrices [66]. Acidic EPS can function as useful material such as water-retention reagent or additive in the fields of food/medical industries [67, 68]. Carbon source selection was found to affect not only the monosaccharide composition of EPS but also their in vitro antioxidant activity and thermogravimetric properties. Among these polysaccharides, FEPS-1 displayed relatively weak antioxidant activity, whereas GEPS-1 showed significantly stronger in vitro antioxidant activity. Notably, GEPS-1 also exhibited good thermal stability, enhancing its potential for food industry applications. Thus, manipulation of culture substrates offers a tractable strategy to tune EPS charge properties for specific functional roles, including fat mimetics, freeze–thaw stabilizers, or emulsifiers in food formulations.

The regulatory responses observed in *P. pentosaceus* LL-07 share both conserved and unique features with other lactic acid bacteria. In *Lactobacillus plantarum* and *Lactobacillus rhamnosus*, carbon source–dependent induction of the Leloir pathway and UDP-sugar biosynthesis has similarly been linked to enhanced EPS production, underscoring a conserved role of precursor supply in LAB [38, 56]. Likewise, the tyrosine kinase–phosphatase module (Wzc/Wze–CpsB/Wzb) is widely distributed across Gram-positive bacteria, including *Streptococcus pneumoniae*, where it regulates capsule assembly through reversible phosphorylation [57, 58]. However, the magnitude of transcriptional reprogramming in *P. pentosaceus* appears more pronounced than in most *Lactobacillus* strains grown on lactose [34], suggesting species-specific regulatory tuning. These comparisons suggest that *P. pentosaceus* LL-07 maintains core LAB strategies for precursor and chain-length control but may have evolved additional transcriptional plasticity to fine-tune EPS output in response to carbon sources.

In summary, our results show that carbon source strongly affects EPS sugar composition through transcriptional control of precursor pathways and GT expression. This control over structural attributes has direct implications for the functional performance of EPS in food, pharmaceutical, and cosmetic applications. Designing fermentation based on substrate-driven gene regulation may help tailor EPS properties for industrial use.

## Conclusion

This study reveals that carbon source selection significantly shapes transcriptional regulation of EPS biosynthesis in *P. pentosaceus* LL-07. RNA-Seq analysis showed that lactose induced extensive upregulation of genes involved in sugar transport, the Leloir pathway, nucleotide sugar biosynthesis, and key EPS gene cluster components. GO and KEGG enrichment analyses linked these transcriptional responses to carbohydrate metabolism, nucleotide sugar formation, and peptidoglycan recycling pathways, indicating tight coordination between primary metabolism and EPS assembly. These changes were accompanied by shifts in EPS monosaccharide composition: lactose promoting galactose-rich EPS, while fructose led to mannose enrichment. Differences in monosaccharide composition caused variations in in vitro antioxidant activity and thermogravimetric properties among purified polysaccharides, suggesting their potential in food industry applications. These findings provide a foundation for substrate-guided engineering of microbial EPS with tailored structural and functional properties. However, the current study has limitations. Subsequent research needs to further validate metabolic flux changes through experiments such as key enzyme activity determination, metabolite quantification, and post-transcriptional regulation investigation. Additionally, the functional implications of EPS composition differences—including bioactivity and physicochemical properties—remain to be explored. Future studies integrating multi-omics approaches and phenotype-function assessments will help optimize EPS production for food, probiotic, and biomedical applications.

## Supplementary Information

Supplementary Material 1.

## Data Availability

The transcriptomic data and P. pentosaceus LL-07 genome sequence were submitted to the GenBank database, and the accession number were PRJNA1288134 and PRJNA904022, respectively. All other data generated are included in the supplemental material files.

## References

[1] Gao Y, Niu M, Yu X, Bao T, Wu Z, Zhao X. Horizontally acquired polysaccharide-synthetic gene cluster from *Weissella cibaria* boosts the probiotic property of *Lactiplantibacillus plantarum*. Front Microbiol. 2021;12:692957. 10.3389/fmicb.2021.692957. 34234766 10.3389/fmicb.2021.692957 PMC8256895

[2] Adebayo-Tayo BC, Ogundele BR, Ajani OA, Olaniyi OA. Characterization of lactic acid bacterium exopolysaccharide, biological, and nutritional evaluation of probiotic formulated fermented coconut beverage. Int J Food Sci. 2024;2024:8923217. 10.1155/2024/8923217. 39257841 10.1155/2024/8923217 PMC11383652

[3] Bibi A, Xiong Y, Rajoka MSR, Mehwish HM, Radicetti E, Umair M, et al. Recent advances in the production of exopolysaccharide (EPS) from *Lactobacillus spp* and its application in the food industry: a review. Sustainability. 2021;13(22):12429. 10.3390/su132212429.

[4] Chen L, Hui YX, Gao TT, Shu G, Chen H. Function and characterization of novel antioxidant peptides by fermentation with a wild Lactobacillus plantarum 60. LWT-food Sci Technol. 2021;135(2):110162. 10.1016/j.lwt.2020.110162.

[5] Ahmed A, Catherine K, Zhang ZQ, Sabra M, Shi G, Tucker A. *Lactobacillus plantarum* probiotic induces Nrf2-mediated antioxidant signaling and eNOS expression resulting in improvement of myocardial diastolic function. Am J PhysiologyHeart Circ Physiol. 2021;321(5):839–49. 10.1152/ajpheart.00278.2021. 10.1152/ajpheart.00278.2021 PMC8616611 34506225

[6] El-Sharkawy H, Tahoun A, Rizk AM, Suzuki T, Elmonir W, Nassef E, et al. Evaluation of *Bifidobacteria* and *Lactobacillus* probiotics as alternative therapy for *Salmonella typhimurium* infection in broiler chickens. Animals. 2020;10(6):1023. 10.3390/ani10061023. 32545606 10.3390/ani10061023 PMC7341506

[7] Akoumany K, Zykwinska A, Sinquin C, Marchand L, Fanuel M, Ropartz D, et al. Characterization of new oligosaccharides obtained by an enzymatic cleavage of the exopolysaccharide produced by the Deep-Sea bacterium *alteromonas infernus* using its cell extract. Molecules. 2019;24(19):3441. 10.3390/molecules24193441. 31546751 10.3390/molecules24193441 PMC6804119

[8] Zhang K, Liu S, Liang S, Xiang F, Wang X, Lian H, et al. Exopolysaccharides of lactic acid bacteria: Structure, biological activity, structure-activity relationship, and application in the food industry: A review. Int J Biol Macromol. 2024;257(Pt 2):128733. 10.1016/j.ijbiomac.2023.128733. 38092118 10.1016/j.ijbiomac.2023.128733

[9] Werren JP, Troxler LJ, Oyewole OR, Ramette A, Brugger SD, Bruggmann R, et al. Carbon source-dependent changes of the structure of *Streptococcus pneumoniae* capsular polysaccharide with serotype 6F. Int J Mol Sci. 2021;22(9):4580. 10.3390/ijms22094580. 33925509 10.3390/ijms22094580 PMC8123889

[10] German B, Schiffrin EJ, Reniero R, Mollet B, Pfeifer A, Neeser JR. The development of functional foods: lessons from the gut. Trends Biotechnol. 1999;17:492–9. 10.1016/s0167-7799(99)01380-3. 10557163 10.1016/s0167-7799(99)01380-3

[11] Cerning J, Renard CM, Thibault JF, Bouillanne C, Landon M, Desmazeaud M, et al. Carbon source requirements for exopolysaccharide production by *Lactobacillus casei* CG11 and partial structure analysis of the polymer. Appl Environ Microbiol. 1994;60(11):3914–9. 10.1128/aem.60.11.3914-3919.1994. 16349427 10.1128/aem.60.11.3914-3919.1994 PMC201915

[12] Angelov A, Georgieva A, Petkova M, Bartkiene E, Rocha JM, Ognyanov M, et al. On the molecular selection of exopolysaccharide-producing lactic acid bacteria from Indigenous fermented plant-based foods and further fine chemical characterization. Foods. 2023;12(18):3346. 10.3390/foods12183346. 37761055 10.3390/foods12183346 PMC10527965

[13] Valerio F, Bavaro AR, Biase MD, Lonigro SL, Logrieco AF, Lavermicocca P. Effect of amaranth and quinoa flours on exopolysaccharide production and protein profile of liquid sourdough fermented by *Weissella cibaria* and *Lactobacillus plantarum*. Front Microbiol. 2020;11:967. 10.3389/fmicb.2020.00967. 32508785 10.3389/fmicb.2020.00967 PMC7253592

[14] Wang G, Li J, Xie S, Zhai Z, Hao Y. The N-terminal domain of rhamnosyltransferase EpsF influences exopolysaccharide chain length determination in *Streptococcus thermophilus* 05–34. Peer J. 2020;12:8e8524. 10.7717/peerj.8524. 10.7717/peerj.8524 PMC7023835 32095353

[15] Wu J, Han X, Ye M, Li Y, Wang X, Zhong Q. Exopolysaccharides synthesized by lactic acid bacteria: biosynthesis pathway, structure-function relationship, structural modification and applicability. Crit Rev Food Sci Nutr. 2023;63(24):7043–64. 10.1080/10408398.2022.2043822. 35213280 10.1080/10408398.2022.2043822

[16] Li M, Li W, Li D, Tian J, Xiao L, Kwok L, et al. Structure characterization, antioxidant capacity, rheological characteristics and expression of biosynthetic genes of exopolysaccharides produced by *Lactococcus lactis* subsp. *lactis* IMAU11823. Food Chem. 2022;384:132566. 10.1016/j.foodchem.2022.132566. 35247774 10.1016/j.foodchem.2022.132566

[17] Zhang J, Xiao Y, Wang H, Zhang H, Chen W, Lu W. Lactic acid bacteria-derived exopolysaccharide: Formation, immunomodulatory ability, health effects, and structure-function relationship. Microbiol Res. 2023;274:127432. 10.1016/j.micres.2023.127432. 37320895 10.1016/j.micres.2023.127432

[18] Siezen RJ, Bayjanov JR, Felis GE, Sijde MR, Starrenburg M, Molenaar D, et al. Genome-scale diversity and niche adaptation analysis of *Lactococcus lactis* by comparative genome hybridization using multi-strain arrays. Microb Biotechnol. 2011;4(3):383–402. 10.1111/j.1751-7915.2011.00247.x. 21338475 10.1111/j.1751-7915.2011.00247.x PMC3818997

[19] Xu X, Bi S, Lao T, Chen F, Liao X, Wu J. Comprehensive investigation on volatile and non-volatile metabolites in broccoli juices fermented by animal- and plant-derived *Pediococcus Pentosaceus*. Food Chem. 2020;341:128118. 10.1016/j.foodchem.2020.128118. 33022577 10.1016/j.foodchem.2020.128118

[20] Odutayo OE, Omonigbehin EA, Olawole TD, Ogunlana OO, Afolabi IS. Fermentation enhanced biotransformation of compounds in the kernel of *Chrysophyllum albidum*. Molecules. 2020;25:6021. 10.3390/molecules25246021. 33352625 10.3390/molecules25246021 PMC7768532

[21] Abouelela ME, Helmy YA. Next-generation probiotics as novel therapeutics for improving human health: current trends and future perspectives. Microorganisms. 2024;12(3):430. 10.3390/microorganisms12030430. 38543481 10.3390/microorganisms12030430 PMC10972033

[22] Bian X, Yang L, Wu W, Lv L, Jiang X, Wang Q, et al. *Pediococcus Pentosaceus* LI05 alleviates DSS-induced colitis by modulating immunological profiles, the gut microbiota and short‐chain fatty acid levels in a mouse model. Microb Biotechnol. 2020;13(4):1228–44. 10.1111/1751-7915.13583. 32363766 10.1111/1751-7915.13583 PMC7264873

[23] Dong F, Xiao F, Li X, Li Y, Wang X, Yu G, et al. *Pediococcus pentosaceus* CECT 8330 protects DSS-induced colitis and regulates the intestinal microbiota and immune responses in mice. J Transl Med. 2022;20:33. 10.1186/s12967-022-03235-8. 35033121 10.1186/s12967-022-03235-8 PMC8761308

[24] Lu K, Wang X, Zhou Y, Zhu Q. Genomic characterization and probiotic potential assessment of an exopolysaccharide-producing strain *Pediococcus pentosaceus* LL-07 isolated from fermented meat. BMC Microbiol. 2024;24:142. 10.1186/s12866-024-03304-6. 38664612 10.1186/s12866-024-03304-6 PMC11044368

[25] DuBois M, Gilles KA, Hamilton JK, Rebers PA, Smith F. Colorimetric method for determination of sugars and related substances. Anal Chem. 1956;28:350–6. 10.1021/ac60111a017.

[26] Klopfenstein DV, Zhang L, Pedersen BS, Ramírez F, Vesztrocy AW, Naldi A, et al. Goatools: a python library for gene ontology analyses. Sci Rep. 2018;8(1):10872. 10.1038/s41598-018-28948-z. 30022098 10.1038/s41598-018-28948-z PMC6052049

[27] Bu D, Luo H, Huo P, Wang Z, Zhang S, He Z, et al. KOBAS-i: intelligent prioritization and exploratory visualization of biological functions for gene enrichment analysis. Nucleic Acids Res. 2021;49(W1):W317–25. 10.1093/nar/gkab447. 34086934 10.1093/nar/gkab447 PMC8265193

[28] Marklund S, Marklund G. Involvement of the superoxide anion radical in the autoxidation of pyrogallol and a convenient assay for superoxide dismutase. Eur J Biochem. 1974;47(3):469–74. 10.1111/j.1432-1033.1974.tb03714.x. 4215654 10.1111/j.1432-1033.1974.tb03714.x

[29] Valu MV, Soare LC, Sutan NA, Ducu C, Moga S, Hritcu L, et al. Optimization of ultrasonic extraction to obtain erinacine A and polyphenols with antioxidant activity from the fungal biomass of hericium Erinaceus. Foods. 2020;9(12):1889. 10.3390/foods9121889. 33352839 10.3390/foods9121889 PMC7766035

[30] Ma B, Wang J, Xu C, Wang Z, Yin D, Zhou B, et al. Interrelation analysis between phenolic compounds and in vitro antioxidant activities in Pu-erh tea. LWT. 2022;158:113117. 10.1016/j.lwt.2022.113117.

[31] Monsan P, Bozonnet S, Albenne C, Joucla G, Willemot RM, Remaud-Siméon M. Homopolysaccharides from lactic acid bacteria. Int Dairy J. 2001;11(9):675–85. 10.1016/S0958-6946(01)00113-3.

[32] Li N, Huang Y, Liu Z, You C, Guo B. Regulation of EPS production in *Lactobacillus casei* LC2W through metabolic engineering. Lett Appl Microbiol. 2015;61(6):555–61. 10.1111/lam.12492. 26370507 10.1111/lam.12492

[33] Wu SC, Kamili NA, Dias-Baruffi M, Josephson CD, Rathgeber MF, Yeung MY, et al. Innate immune Galectin-7 specifically targets microbes that decorate themselves in blood group-like antigens. iScience. 2022;25(7):104482. 10.1016/j.isci.2022.104482. 35754739 10.1016/j.isci.2022.104482 PMC9218387

[34] Bamigbade G, Ali AH, Subhash A, Tamiello-Rosa C, Qudsi FRA, Esposito G, et al. Structural characterization, biofunctionality, and environmental factors impacting rheological properties of exopolysaccharide produced by probiotic *Lactococcus lactis* C15. Sci Rep. 2023;13(1):17888. 10.1038/s41598-023-44728-w. 37857676 10.1038/s41598-023-44728-w PMC10587178

[35] Hu H, Peng Q, Tai J, Lu W, Liu J, Dan T. Unveiling the genetic basis and metabolic rewiring behind the galactose-positive phenotype in a *Streptococcus thermophilus* mutant. Microbiol Res. 2024;289:127894. 10.1016/j.micres.2024.127894. 39305781 10.1016/j.micres.2024.127894

[36] Schroeder CJ, Robert C, Lenzen G, McKay LL, Mercenier A. Analysis of the LacZ sequences from two *Streptococcus thermophilus* strains: comparison with the *Escherichia coli* and *Lactobacillus bulgaricus* beta-galactosidase sequences. Microbiology. 1991;137:369–80. 10.1099/00221287-137-2-369. 10.1099/00221287-137-2-369 1901904

[37] Foucaud C, Poolman B. Lactose transport system of *Streptococcus thermophilus*. Functional reconstitution of the protein and characterization of the kinetic mechanism of transport. J Biol Chem. 1992;267:22087–94. 10.1016/S0021-9258(18)41639-0. 1429561

[38] Rodríguez J, Vázquez L, Flórez AB, Mayo B. Phenotype testing, genome analysis, and metabolic interactions of three lactic acid bacteria strains existing as a consortium in a naturally fermented milk. Front Microbiol. 2022;13:1000683. 10.3389/fmicb.2022.1000683. 36212860 10.3389/fmicb.2022.1000683 PMC9539746

[39] Lin S, Wu T, Lin H, Zhang Y, Xu S, Wang J, et al. De novo analysis reveals transcriptomic responses in *Eriobotrya Japonica* fruits during postharvest cold storage. Genes. 2018;9(12):639. 10.3390/genes9120639. 30563027 10.3390/genes9120639 PMC6316545

[40] Pastor JM, Borges N, Pagán JP, Castaño-Cerezo S, Csonka LN, Goodner BW, et al. Fructose metabolism in *Chromohalobacter salexigens*: interplay between the Embden-Meyerhof-Parnas and Entner-Doudoroff pathways. Microb Cell Fact. 2019;18(1):134. 10.1186/s12934-019-1178-x. 31409414 10.1186/s12934-019-1178-x PMC6692947

[41] Holowachuk EW, Friesen JD, Fiil NP. *Bacteriophage lambda* vehicle for the direct cloning of *Escherichia coli* promoter DNA sequences: feedback regulation of the rplJL-rpoBC operon. Proc Natl Acad Sci USA. 1980;77(4):2124–8. 10.1073/pnas.77.4.2124. 6445564 10.1073/pnas.77.4.2124 PMC348664

[42] Mori M, Marinari E, Martino AD. A yield-cost tradeoff governs *Escherichia coli*’s decision between fermentation and respiration in carbon-limited growth. NPJ Syst Biol Appl. 2019;5:16. 10.1038/s41540-019-0093-4. 31069113 10.1038/s41540-019-0093-4 PMC6494807

[43] Coelho AI, Trabuco M, Ramos R, Silva MJ, de Almeida IT, Leandro P, et al. Functional and structural impact of the most prevalent missense mutations in classic galactosemia. Mol Genet Genomic Med. 2014;2(6):484–96. 10.1002/mgg3.94. 25614870 10.1002/mgg3.94 PMC4303218

[44] Xing Z, Geng W, Li C, Sun Y, Wang Y. Comparative genomics of *Lactobacillus kefiranofaciens* ZW3 and related members of *Lactobacillus*. spp reveal adaptations to dairy and gut environments. Sci Rep. 2017;7(1):12827. 10.1038/s41598-017-12916-0. 28993659 10.1038/s41598-017-12916-0 PMC5634458

[45] Fu L, Jiang B, Wei J, Liu J, Hu X, Zhang L. Transcriptome analysis of polysaccharide-based microbial flocculant MBFA9 biosynthesis regulated by nitrogen source. Sci Rep. 2020;10(1):2918. 10.1038/s41598-020-59114-z. 32075995 10.1038/s41598-020-59114-z PMC7031244

[46] Levander F, Svensson M, Rådström P. Enhanced exopolysaccharide production by metabolic engineering of *Streptococcus thermophilus*. Appl Environ Microbiol. 2002;68(2):784–90. 10.1128/AEM.68.2.784-790.2002. 11823219 10.1128/AEM.68.2.784-790.2002 PMC126717

[47] Giordano I, Pasolli E, Mauriello G. Transcriptomic analysis reveals differential gene expression patterns of *Lacticaseibacillus casei* ATCC 393 in response to ultrasound stress. Ultrason Sonochem. 2024;107:106939. 10.1016/j.ultsonch.2024.106939. 38843696 10.1016/j.ultsonch.2024.106939 PMC11214525

[48] Han Y, Luo P, Zeng H, Wang P, Xu J, Chen P, et al. The effect of O-antigen length determinant *Wzz* on the immunogenicity of *Salmonella typhimurium* for *Escherichia coli* O2 O-polysaccharides delivery. Vet Res. 2023;54(1):15. 10.1186/s13567-023-01142-4. 36849993 10.1186/s13567-023-01142-4 PMC9969949

[49] Pardo-Esté C, Castro-Severyn J, Krüger GI, Cabezas CE, Briones AC, Aguirre C, et al. The transcription factor ArcA modulates *Salmonella*’s metabolism in response to neutrophil hypochlorous acid-mediated stress. Front Microbiol. 2019;10:2754. 10.3389/fmicb.2019.02754. 31866961 10.3389/fmicb.2019.02754 PMC6906141

[50] Woodward R, Yi W, Li L, Zhao G, Eguchi H, Sridhar PR, et al. *In vitro* bacterial polysaccharide biosynthesis: defining the functions of Wzy and Wzz. Nat Chem Biol. 2010;6(6):418–23. 10.1038/nchembio.351. 20418877 10.1038/nchembio.351 PMC2921718

[51] Zhang M, Lai T, Yao M, Zhang M, Yang Z. Interaction of the exopolysaccharide from *Lactobacillus plantarum* YW11 with casein and bioactivities of the polymer complex. Foods. 2021;10(6):1153. 10.3390/foods10061153. 34063954 10.3390/foods10061153 PMC8224047

[52] Yang Yun, Liu J, Clarke BR, Seidel L, Bolla JR, Ward PN. The molecular basis of regulation of bacterial capsule assembly by Wzc. Nat Commun. 2021;12(1):4349. 10.1038/s41467-021-24652-1. 34272394 10.1038/s41467-021-24652-1 PMC8285477

[53] Bender MH, Cartee RT, Yother J. Positive correlation between tyrosine phosphorylation of CpsD and capsular polysaccharide production in *Streptococcus pneumoniae*. J Bacteriol. 2003;185(20):6057–66. 10.1128/JB.185.20.6057-6066.2003. 14526017 10.1128/JB.185.20.6057-6066.2003 PMC225014

[54] Maajid HS, Nurliyani N, Widodo W. Exopolysaccharide production in fermented milk using *Lactobacillus casei* strains AP and AG. AIMS Microbiol. 2022;8(2):138–52. 10.3934/microbiol.2022012. 35974991 10.3934/microbiol.2022012 PMC9329877

[55] Guérin H, Courtin P, Guillot A, Péchoux C, Mahony J, Sinderen D, et al. Molecular mechanisms underlying the structural diversity of rhamnose-rich cell wall polysaccharides in Lactococci. J Biol Chem. 2024;300(1):105578. 10.1016/j.jbc.2023.105578. 38110036 10.1016/j.jbc.2023.105578 PMC10821137

[56] Kang H, Gilbert C, Badeaux F, Atlan D, LaPointe G. A tyrosine phosphorylation switch controls the interaction between the transmembrane modulator protein Wzd and the tyrosine kinase Wze of *Lactobacillus rhamnosus*. BMC Microbiol. 2015;15:40. 10.1186/s12866-015-0371-2. 25885688 10.1186/s12866-015-0371-2 PMC4340800

[57] Nourikyan J, Kjos M, Mercy C, Cluzel C, Morlot C, Noirot-Gros MF, et al. Autophosphorylation of the bacterial tyrosine-kinase CpsD connects capsule synthesis with the cell cycle in *Streptococcus pneumoniae*. PLoS Genet. 2015;11(9):e1005518. 10.1371/journal.pgen.1005518. 26378458 10.1371/journal.pgen.1005518 PMC4574921

[58] Toniolo C, Balducci E, Romano MR, Proietti D, Ferlenghi I, Grandi G, et al. *Streptococcus* agalactiae capsule polymer length and attachment is determined by the proteins CpsABCD. J Biol Chem. 2015;290(15):9521–32. 10.1074/jbc.M114.631499. 25666613 10.1074/jbc.M114.631499 PMC4392257

[59] Sørensen HM, Rochfort KD, Maye S, MacLeod G, Brabazon D, Loscher C, et al. Exopolysaccharides of lactic acid bacteria: production, purification and health benefits towards functional food. Nutrients. 2022;14(14):2938. 10.3390/nu14142938. 35889895 10.3390/nu14142938 PMC9319976

[60] Fuso A, Bancalari E, Castellone V, Caligiani A, Gatti M, Bottari B. Feeding lactic acid bacteria with different sugars: effect on exopolysaccharides (EPS) production and their molecular characteristics. Foods. 2023;12(1):215. 10.3390/foods12010215. 36613431 10.3390/foods12010215 PMC9819028

[61] Maitra A, Munshi T, Healy J, Martin LT, Vollmer W, Keep NH, et al. Cell wall peptidoglycan in *Mycobacterium tuberculosis*: an achilles’ heel for the TB-causing pathogen. FEMS Microbiol Rev. 2019;43(5):548–75. 10.1093/femsre/fuz016. 31183501 10.1093/femsre/fuz016 PMC6736417

[62] Xia X, Li J, Zhou Z, Wang D, Huang J, Wang G. High-quality-draft genome sequence of the multiple heavy metal resistant bacterium *Pseudaminobacter manganicus* JH-7T. Stand Genomic Sci. 2018;13:29. 10.1186/s40793-018-0330-2. 30386456 10.1186/s40793-018-0330-2 PMC6203287

[63] Li L, Li H, Qian J, He Y, Zheng J, Lu Z, et al. Structural and immunological activity characterization of a polysaccharide isolated from *Meretrix meretrix* Linnaeus. Mar Drugs. 2015;14(1):6. 10.3390/md14010006. 26729136 10.3390/md14010006 PMC4728503

[64] Jaroentomeechai T, Kwon YH, Liu Y, Young O, Bhawal R, Wilson JD, et al. A universal glycoenzyme biosynthesis pipeline that enables efficient cell-free remodeling of glycans. Nat Commun. 2022;13(1):6325. 10.1038/s41467-022-34029-7. 36280670 10.1038/s41467-022-34029-7 PMC9592599

[65] Hollander N, Haimovich J. Altered. N-linked glycosylation in follicular lymphoma and chronic lymphocytic leukemia: involvement in pathogenesis and potential therapeutic targeting. Front Immunol. 2017;8:912. 10.3389/fimmu.2017.00912. 28824637 10.3389/fimmu.2017.00912 PMC5539419

[66] Surber G, Jaros D, Rohm H. Shear and extensional rheology of acid milk gel suspensions with varying ropiness. J Texture Stud. 2020;51(1):111–9. 10.1111/jtxs.12458. 31226221 10.1111/jtxs.12458

[67] Ngouénam JR, Kenfack CHM, Kouam EMF, Kaktcham PM, Maharjan R, Ngoufack FZ. Lactic acid production ability of *Lactobacillus* sp. from four tropical fruits using their by-products as carbon source. Heliyon. 2021;7(5):e07079. 10.1016/j.heliyon.2021.e07079. 34136681 10.1016/j.heliyon.2021.e07079 PMC8180611

[68] Ngatu NR, Okajima MK, Yokogawa M, Hirota R, Eitoku M, Muzembo BA et al. Anti-inflammatory effects of sacran, a novel polysaccharide from *Aphanothece sacrum*, on 2,4,6-trinitrochlorobenzene-induced allergic dermatitis *in vivo*. Annals of allergy, asthma, and immunology. 2012;108(2):117–22. 10.1016/j.anai.2011.10.013 10.1016/j.anai.2011.10.013 22289731

