# Supplementary material for: Carbon source–dependent transcriptomic regulation and monosaccharide remodeling of exopolysaccharide biosynthesis in Pediococcus pceusentosa LL-07
Source: BMC Microbiol. 2025 Oct 28;25:695. doi: 10.1186/s12866-025-04409-2 (PMC12570439; doi:10.1186/s12866-025-04409-2)
Supplement: Supplementary file 1 — Supplementary Material 1. [file 12866_2025_4409_MOESM1_ESM.pdf]

Supplementary Materials

Carbon Source–Dependent Transcriptomic Regulation and Monosaccharide Remodeling of Exopolysaccharide Biosynthesis in *Pediococcus pentosaceus* LL-07

Kuan Lu[a,b], Xueya Wang[c], Ying Zhou[a*] , Qiujin Zhu[a*]

[a] Guizhou Province Key Laboratory of Agricultural and Animal Products Storage and Processing, School of Liquor and Food Engineering, Guizhou University, Guizhou, Guiyang, 550025, China
[b] Guizhou Biotechnology Research and Development Base Co., Ltd., Guizhou, Guiyang, 550025, China
[c] Chili Pepper Research Institute, Guizhou Provincial Academy of Agricultural Sciences, Guizhou, Guiyang, 550006, China

* Corresponding author

E-mail address:ls.qjzhu@gzu.edu.cn. zhying_0525@163.com

CONTEST

Supplementary Table 1 The primers used in qRT-PCR 2
Supplementary Table 2 Raw data statistics 3
Supplementary Table 3 Clean data information 4
Supplementary Table 4 RNA-seq comparison statistics of each group 5
Supplementary Table 5 rRNA depletion efficiency and RNA integrity numbers 6
Supplementary Table 6 Half inhibitory concentration of EPS 7
Supplementary Fig.1 Detection spectrum of RNA integrity numbers 8
Supplementary Fig.2 UV-vis spectrum of EPS 9
Supplementary Fig.3 The antioxidant activity of EPS 10
Supplementary Fig.4 Thermogravimetric analysis of EPS 11
Supplementary Fig.5 Ion chromatogram of monosaccharide composition of EPS 12
Supplementary Fig.6 Amplification curves for all target genes 13
Supplementary Fig.7 Melting curves for all target genes 14

Supplementary Table 1 The primers used in qRT-PCR

Gene name	Gene ID		Primer sequence (5`-3`)	Primer efficiencies (%)
dapB	*gene0132*	F	AGGATCTATGGGTCAACAAACC	102.64
		R	AAAACAACGGAGGGGACG	
pbuG	*gene0521*	F	TGTCAGAACAAGGAACAACGG	103.75
		R	CCATCAAGACGCTTCCCAATA	
ade	*gene0522*	F	GCAGTGTTATGGGGTATGGG	100.30
		R	ATGCGAGTAACTTTCGTGGTC	
ropB	*gene1004*	F	ACAGTCAATGATCTCATCCGGG	101.39
		R	ATGGCTGTGAATGTCGGCTT	
atpD	*gene1270*	F	GATGGAACAGATGGACTACAACG	102.78
		R	CATAATCTGGTGGATCACGATG	
purS	*gene1414*	F	GTTAAAGCTGTAACTGTCGG	99.54
		R	TCCTGCTAATCTCAAATCG	
purK	*gene1416*	F	GCGGATTTGCTTGCTGG	106.47
		R	TGATGTAGAATGTGGTGCTGATG	
padR	*gene1762*	F	CTTTCCGATTTCTGAATCAACC	101.00
		R	GCCAAACGAACTTTACCTGCT	
galM	*gene0191*	F	TAAACGCTGAAAATGGTGGG	100.27
		R	TGGTCGGGATCAAATGGG	
galK	*gene0195*	F	TCAAAGAACCATCAAGGCAACT	102.54
		R	AAACCAGCACCAGTCATTCG	
galE	*gene0196*	F	AGGTTACGATGTAGCGGTTGTT	102.76
		R	CGTTTTCTTTGGTGAAGACTGA	
galT	*gene0197*	F	AAAGCGGCGACGGATTA	101.44
		R	CCTGAGGTTGATTACGAGCAG	
pyrE	*gene0297*	F	TAATCGGTGGGGTCGCA	101.80
		R	CTCCATGATCCTTGGGTTTG	
pgk	*gene0445*	F	TTGACTCTTCGTCCAGTTGCT	102.33
		R	AATAATACATCGCCGTCGTTC	
malY	*gene0982*	F	CCACCTTTGGCGGGTTT	104.06
		R	TTGGCAGCAGTAGCATCACA	
pyrF	*gene1617*	F	GCCTAGCAAGGTTGACCAGTA	104.78
		R	CAGGAGTCATCGTCCGTTTT	

The *P. pentosaceus* LL-07 genome sequence has been submitted to the GenBank database, and the accession number was PRJNA904022.

Supplementary Table 2 Raw data statistics

Name	reads	Bases (bp)	Error Rate (%)	Q20 (%)	Q30 (%)
G-1-1	31,205,152	4,711,977,952	0.0327	94.32	90.04
G-1-2	29,164,680	4,403,866,680	0.0322	94.42	90.40
G-1-3	31,682,028	4,783,986,228	0.0321	94.50	90.48
G-2-1	30,662,486	4,630,035,386	0.0321	94.40	90.47
G-2-2	27,833,426	4,202,847,326	0.0332	94.01	89.79
G-2-3	25,499,628	3,850,443,828	0.0313	94.77	90.99
F-1-1	30,746,258	4,642,684,958	0.0308	94.87	91.32
F-1-2	27,171,740	4,102,932,740	0.0308	94.95	91.32
F-1-3	28,013,988	4,230,112,188	0.0310	94.80	91.17
F-2-1	29,138,096	4,399,852,496	0.0325	94.34	90.22
F-2-2	27,903,888	4,213,487,088	0.0313	94.77	90.99
F-2-3	24,641,634	3,720,886,734	0.0323	94.40	90.34
L-1-1	39,019,914	5,892,007,014	0.0333	93.73	89.87
L-1-2	27,130,260	4,096,669,260	0.0338	93.54	89.60
L-1-3	28,444,708	4,295,150,908	0.0333	93.67	89.87
L-2-1	28,696,684	4,333,199,284	0.0314	94.75	90.91
L-2-2	25,947,446	3,918,064,346	0.0338	94.12	89.29
L-2-3	23,758,918	3,587,596,618	0.0312	94.76	91.03

Supplementary Table 3 Clean data information

Name	Reads	Bases (bp)	Error Rate (%)	Q20 (%)	Q30 (%)
G-1-1	30,054,562	3,836,691,823	0.0257	97.01	94.93
G-1-2	28,222,176	3,670,475,946	0.0258	96.96	94.83
G-1-3	30,697,582	3,903,081,791	0.0256	97.06	95.03
G-2-1	29,897,254	3,827,526,517	0.0257	97.00	94.91
G-2-2	27,110,236	3,480,254,605	0.0258	96.98	94.84
G-2-3	24,920,824	3,300,938,675	0.0261	96.87	94.64
F-1-1	30,211,680	3,972,205,242	0.0258	96.99	94.87
F-1-2	26,528,602	3,488,257,537	0.0259	96.96	94.80
F-1-3	27,462,174	3,592,594,510	0.0259	96.94	94.80
F-2-1	28,161,648	3,625,032,396	0.0260	96.92	94.72
F-2-2	27,164,170	3,563,587,590	0.0258	97.00	94.81
F-2-3	23,884,452	3,084,030,047	0.0258	96.95	94.83
L-1-1	38,575,580	5,008,027,906	0.0277	95.88	93.54
L-1-2	26,576,140	3,514,747,317	0.0282	95.66	93.20
L-1-3	27,924,206	3,720,502,994	0.0281	95.71	93.26
L-2-1	27,684,532	3,596,887,394	0.0255	97.13	95.05
L-2-2	23,297,236	3,076,626,346	0.0260	96.92	94.73
L-2-3	23,180,698	3,029,765,391	0.0257	97.00	94.91

Supplementary Table 4 RNA-seq comparison statistics of each group

Name	Total Reads	Mapped Reads	Mapped Ratio (%)	Uniq Mapped Reads	Uniq Mapped Reads Ratio (%)
G-1-1	30,211,680	29,514,416	97.69	28,802,557	95.34
G-1-2	26,528,602	25,965,928	97.88	25,363,887	95.61
G-1-3	27,462,174	26,941,380	98.10	26,248,527	95.58
G-2-1	28,161,648	27,477,672	97.57	26,879,801	95.45
G-2-2	27,164,170	26,629,966	98.03	26,412,767	97.23
G-2-3	23,884,452	23,350,269	97.76	23,164,939	96.99
F-1-1	30,054,562	29,368,816	97.72	29,015,494	96.54
F-1-2	28,222,176	27,501,388	97.45	27,166,766	96.26
F-1-3	30,697,582	29,980,473	97.66	29,624,721	96.51
F-2-1	29,897,254	29,237,004	97.79	29,021,442	97.07
F-2-2	27,110,236	26,421,336	97.46	26,219,334	96.71
F-2-3	24,920,824	24,364,753	97.77	23,613,382	94.75
L-1-1	38,575,580	37,995,712	98.50	37,494,758	97.20
L-1-2	26,576,140	26,073,175	98.11	25,817,975	97.15
L-1-3	27,924,206	27,495,979	98.47	27,251,972	97.59
L-2-1	27,684,532	26,839,825	96.95	26,560,329	95.94
L-2-2	23,297,236	22,569,993	96.88	22,424,276	96.25
L-2-3	23,180,698	22,542,550	97.25	22,360,799	96.46

Supplementary Table 5 rRNA depletion efficiency and RNA integrity numbers

Sample name	OD260/280	OD260/230	RIN	rRNA Ratio(%) of Rfam
G1_1	2.20	2.44	10.00	1.804
G1_2	2.19	2.48	10.00	1.959
G1_3	2.18	2.46	10.00	2.007
G2_1	2.16	2.44	10.00	1.578
G2_2	2.19	2.47	10.00	1.674
G2_3	2.20	2.47	10.00	2.863
F1_1	2.18	2.48	10.00	2.603
F1_2	2.18	2.44	10.00	2.433
F1_3	2.19	2.45	10.00	2.866
F2_1	2.18	2.39	10.00	3.262
F2_2	2.18	2.45	10.00	1.27
F2_3	2.19	2.45	10.00	1.351
L1_1	2.21	2.24	9.20	1.066
L1_2	2.09	2.23	9.80	1.156
L1_3	2.21	2.19	9.30	0.875
L2_1	2.10	2.20	10.00	2.096
L2_2	2.11	2.21	10.00	1.76
L2_3	2.09	2.22	10.00	1.818

Supplementary Table 6 Half inhibitory concentration of EPS

Sample ID	IC_{50} (mg/mL)		
	Hydroxyl radical	DPPH radical	Superoxide anion radical
GEPS-1	3.834	4.448	3.352
GEPS-2	7.348	5.36	6.239
GEPS-3	8.294	4.58	12.331
LEPS-1	4.926	5.446	6.799
LEPS-2	11.288	6.321	11.286
LEPS-3	7.973	9.271	10.940
FEPS-1	27.746	8.061	11.730
FEPS-2	14.105	9.908	6.645

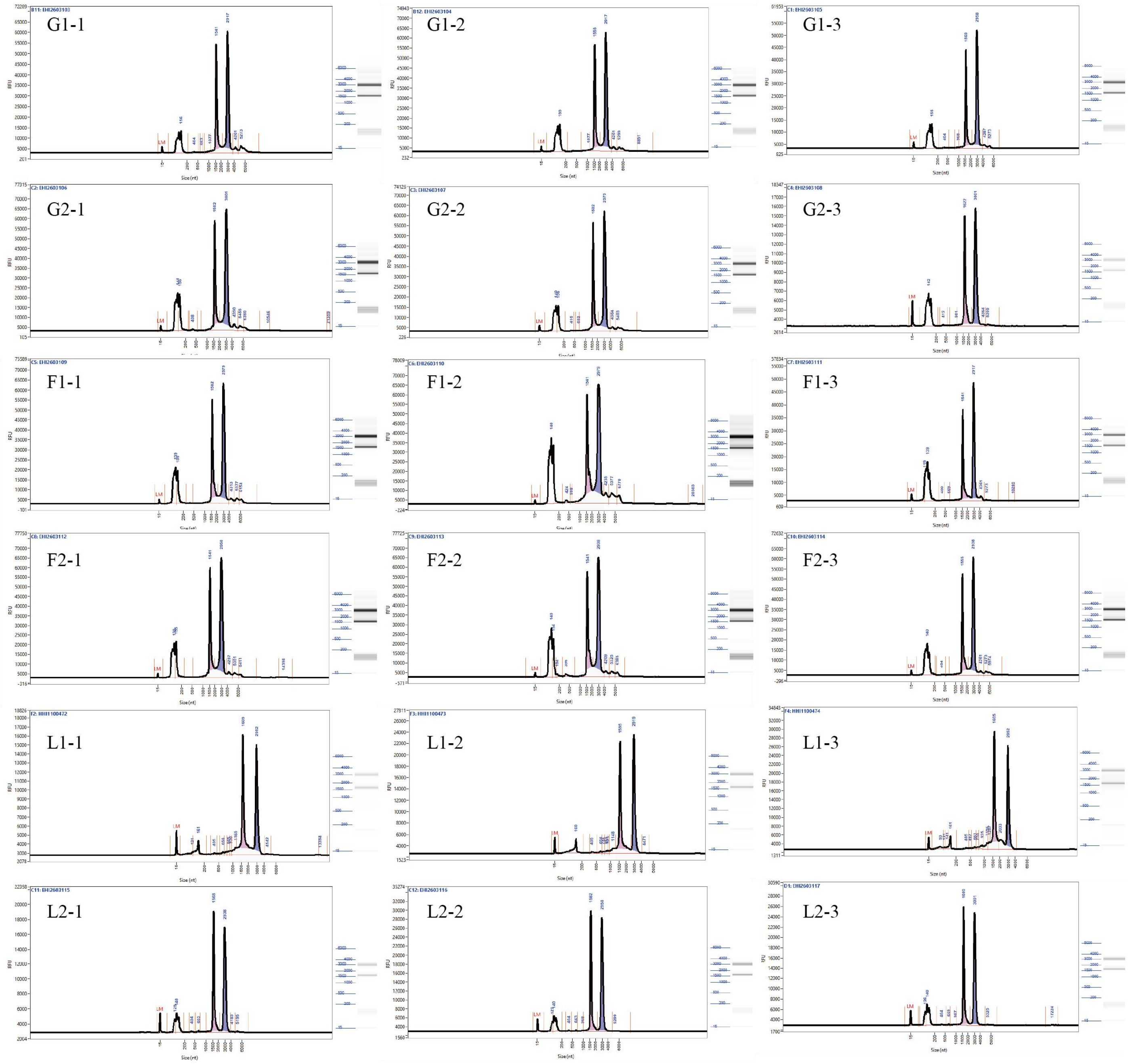

Supplementary Fig.1 Detection spectrum of RNA integrity numbers

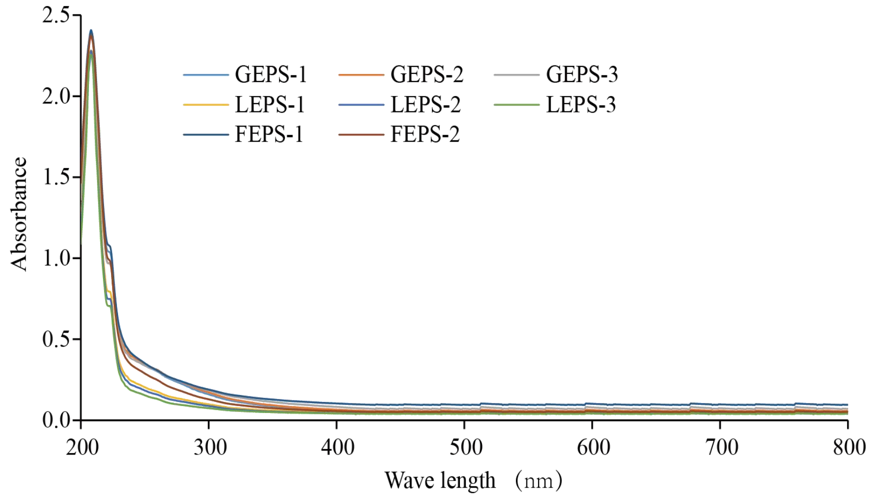

Supplementary Fig.2 UV-vis spectrum of EPS

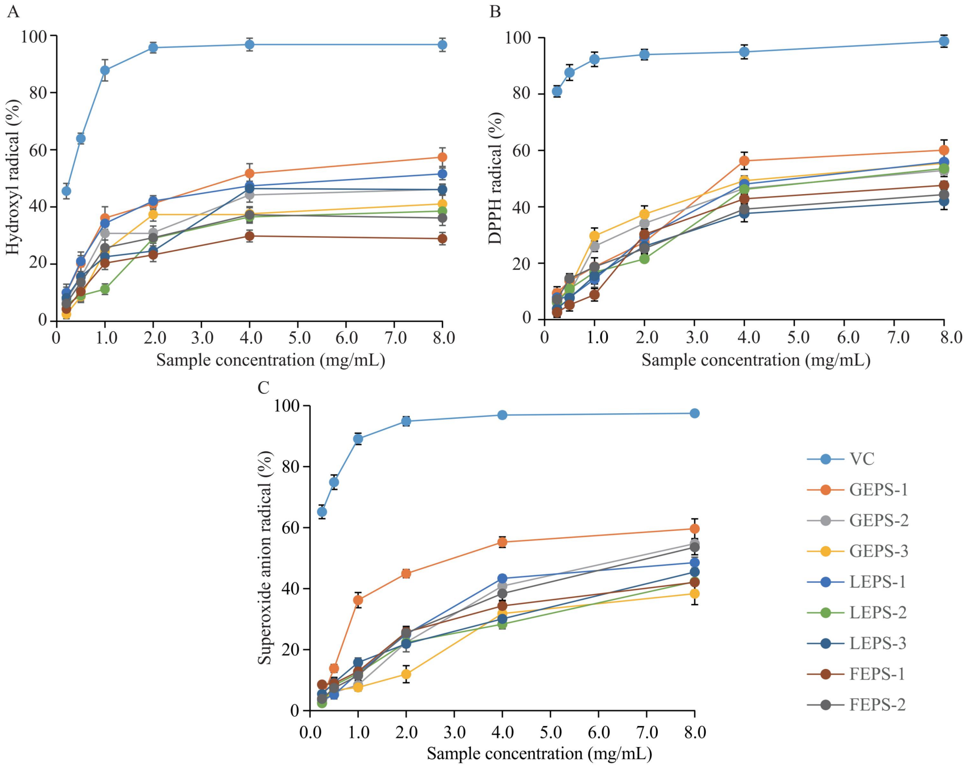

Supplementary Fig.3 The antioxidant activity of EPS

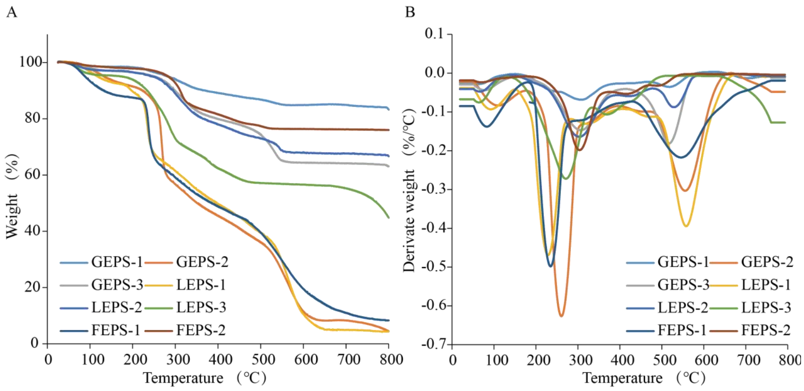

Supplementary Fig.4 Thermogravimetric analysis of EPS. A is the TG curve and B is the DTG curve.

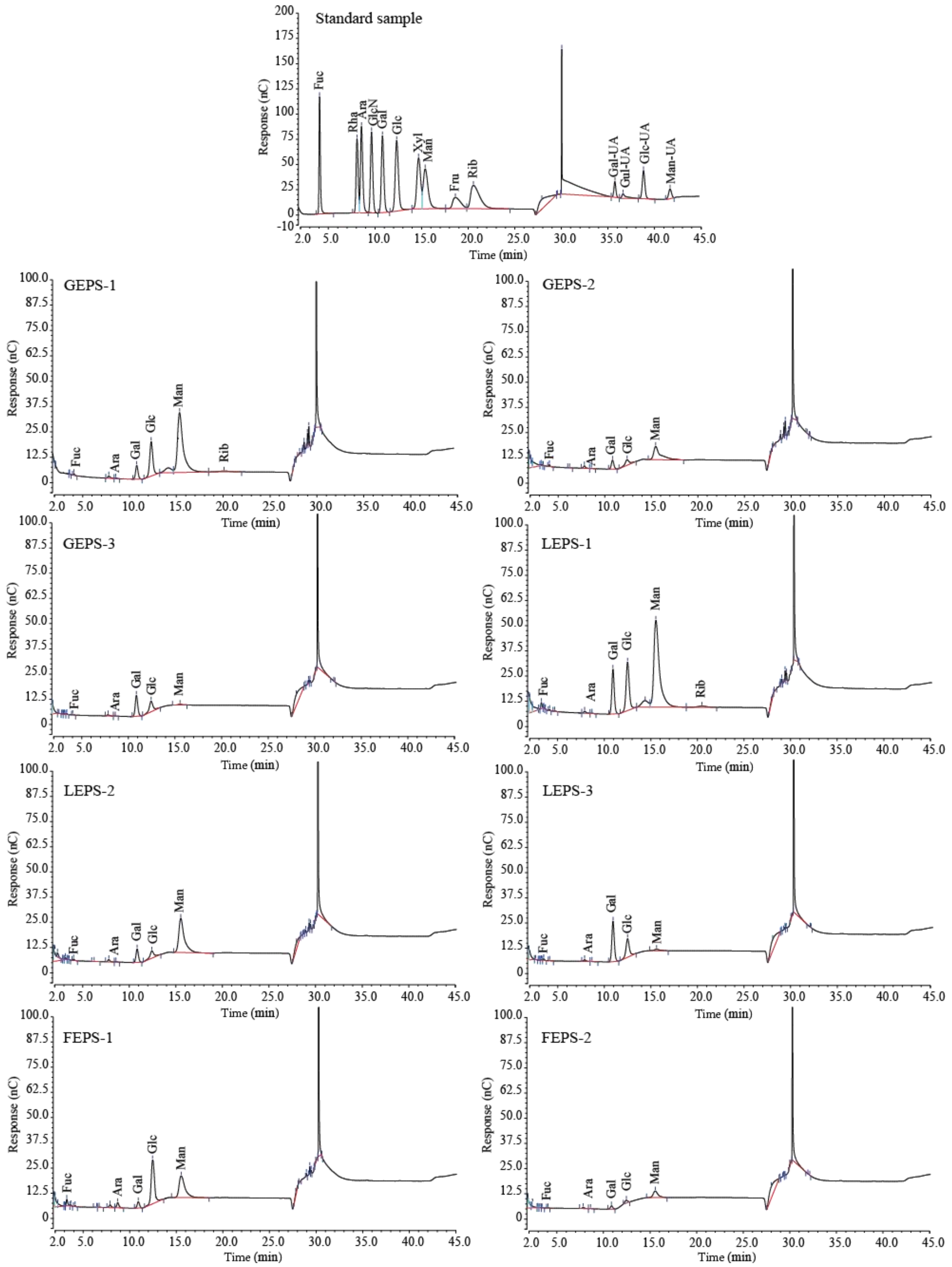

Supplementary Fig.5 Ion chromatogram of monosaccharide composition of EPS

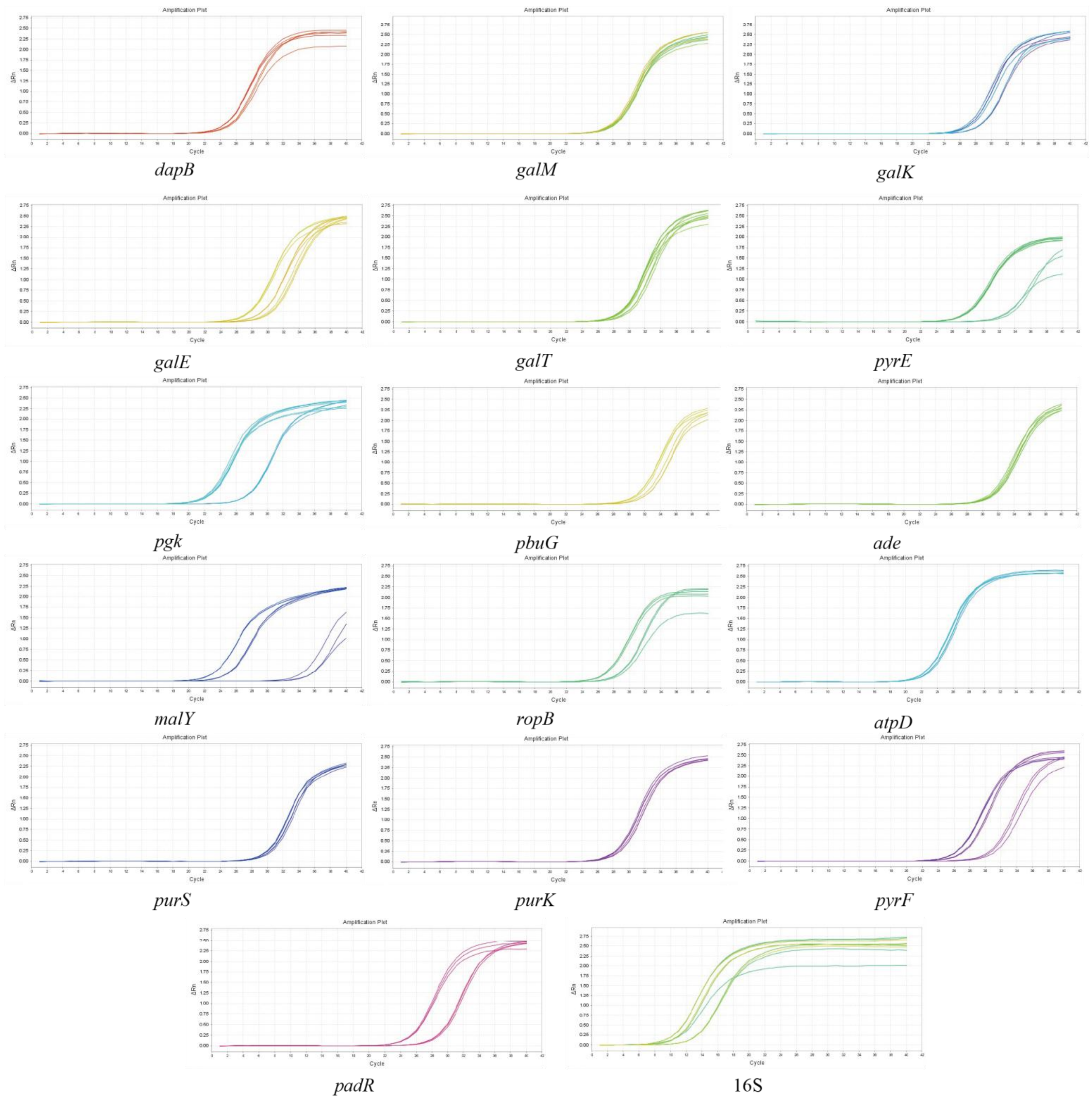

Supplementary Fig.6 Amplification curves for all target genes. The horizontal axis represents △Rn, and the vertical axis represents cycle.

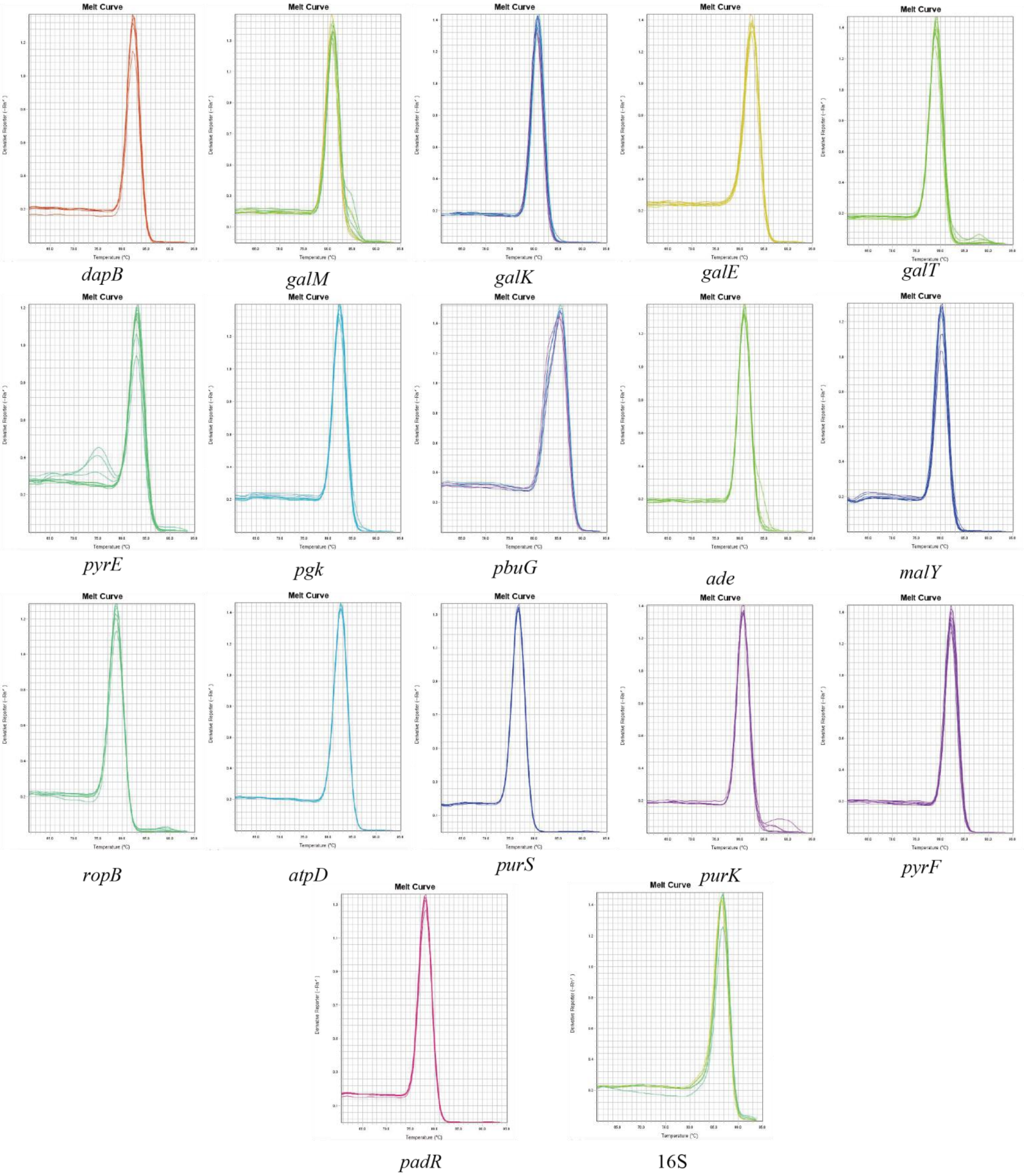

Supplementary Fig.7 Melting curves for all target genes
